# Elucidation of Sequence–Function Relationships for an Improved Biobutanol *In Vivo* Biosensor in *E. coli*

**DOI:** 10.3389/fbioe.2022.821152

**Published:** 2022-02-21

**Authors:** Nancy M. Kim, Riley W. Sinnott, Lily N. Rothschild, Nicholas R. Sandoval

**Affiliations:** ^1^ Interdisciplinary Bioinnovation PhD Program, Tulane University, New Orleans, LA, United States; ^2^ Department of Chemical & Biomolecular Engineering, Tulane University, New Orleans, LA, United States

**Keywords:** promoter engineering, biobutanol, BmoR, sort-seq, transcription factor

## Abstract

Transcription factor (TF)–promoter pairs have been repurposed from native hosts to provide tools to measure intracellular biochemical production titer and dynamically control gene expression. Most often, native TF–promoter systems require rigorous screening to obtain desirable characteristics optimized for biotechnological applications. High-throughput techniques may provide a rational and less labor-intensive strategy to engineer user-defined TF–promoter pairs using fluorescence-activated cell sorting and deep sequencing methods (sort-seq). Based on the designed promoter library’s distribution characteristics, we elucidate sequence–function interactions between the TF and DNA. In this work, we use the sort-seq method to study the sequence–function relationship of a σ^54^-dependent, butanol-responsive TF–promoter pair, BmoR-P_BMO_ derived from *Thauera butanivorans*, at the nucleotide level to improve biosensor characteristics, specifically an improved dynamic range. Activities of promoters from a mutagenized P_BMO_ library were sorted based on *gfp* expression and subsequently deep sequenced to correlate site-specific sequences with changes in dynamic range. We identified site-specific mutations that increase the sensor output. Double mutant and a single mutant, CA(129,130)TC and G(205)A, in P_BMO_ promoter increased dynamic ranges of 4-fold and 1.65-fold compared with the native system, respectively. In addition, sort-seq identified essential sites required for the proper function of the σ^54^-dependent promoter biosensor in the context of the host. This work can enable high-throughput screening methods for strain development.

## Introduction

Cells can monitor and respond to a wide range of fluctuations in their environment and the ability to coordinate rapid and finely tuned responses. Due to this ability, these systems have been engineered to be used as information processing circuits *in vivo* for industrial applications. Ligand-responsive transcriptional regulators have been valuable tools in constructing complex metabolic pathways to increase metabolite concentrations (Kim et al., 2020). They can allow microorganisms to monitor levels of internal and/or external metabolites and adjust gene expression levels to balance pathway function (Zhang et al., 2012; Rohlhill et al., 2017; Tan and Prather, 2017). Furthermore, coupling transcription factor (TF)–based biosensors with fluorescent gene reporters such as GFP screened via fluorescence-activated cell sorting (FACS) has proven to be an efficient high-throughput strategy to accelerate the identification of high producers and fill in gaps of knowledge in metabolic pathways (Zeng et al., 2020).

Current chromatographic methods such as liquid or gas chromatography are low throughput, measuring 10^2^ samples per day at best, while genome-wide libraries are typically several orders of magnitude higher (Dietrich et al., 2010; Sandoval et al., 2012; Bott, 2015). TF-based biosensors are especially useful in high-throughput screening strategies in optimizing and engineering these systems (Lin et al., 2017; Bowman et al., 2021). However, the versatility and the labor-intensive optimization of these biosensors for different applications limit their use. Therefore, there is a need for efficient workflows to explore and optimize these systems. Building reliable tools to monitor product formation requires the ability to discriminate small changes in molecule concentration. Detection of the metabolite of interest requires the biosensor to be able to detect at a lower level than its relevant lowest concentration (lower limit of detection) and higher than its relevant highest concentration (upper limit of detection). Therefore, biosensor properties are evaluated based on four parameters: 1) basal leakage in the absence of the metabolite, 2) the fold-change in the expression at the maximum output relative to the basal activity known as the dynamic range, 3) the concentration of the metabolite required to elicit a 50% response known as the response threshold, and 4) sensitivity to the metabolite of interest (Mannan et al., 2017). Often two main strategies are employed to alter these parameters of a biosensor: 1) generation of a diverse library at the DNA level and 2) combining different components or genetic elements, also known as “parts,” of a system to create a novel system (Levo and Segal, 2014; Cazier and Blazeck, 2021). However, to assay every possible sequence of interest, or combination, would be laborious and difficult to manage the amount of data generated. Furthermore, regulatory elements such as promoters can exhibit different activities in a context-dependent manner, making it difficult to transfer between different hosts.

Massively parallel reporter assays (MPRAs), specifically sort-seq, have emerged as a popular tool used to dissect the gene regulation of promoters by studying sequence specificity of the regulating protein to decipher sequence–function relationships in a quantitative manner (Kinney et al., 2010; Sharon et al., 2012; Sharon et al., 2014; Levy et al., 2017; Rohlhill et al., 2017). The functions of thousands to millions of sequences of specific genetic elements are measured in a single experiment in the context of the host cell, and subsequently sequenced using deep sequencing to build quantitative models to predict the activity of any designed sequence. Measuring the causal effects of mutations on gene expression allows the determinants of TF–DNA binding to be calculated to create functional learning models using statistical inference methods such as mutual information maximization (Kinney et al., 2010).

Biofuels is an active area of research pursued in metabolic engineering endeavors. Biosensors to detect biofuel molecules, specifically butanol, have been developed in *E. coli*. An alcohol-regulated TF BmoR and its cognate promoter P_BMO_ involved in *n*-alkane metabolism of *Thauera butanivorans* (née *Pseudomonas butanovora*) (Kurth et al., 2008) was first demonstrated as a biosensor in *E. coli* to screen for strains with high conversion rate from 2-oxopentoanoate to *n*-butanol (Dietrich et al., 2013). Coupling butanol concentration to cell growth and fluorescence by regulating expression of *tetA* fused with *gfp* gene isolated strains exhibiting butanol synthesizes 120 times higher prior to optimization (Dietrich et al., 2013).

**FIGURE 1:**
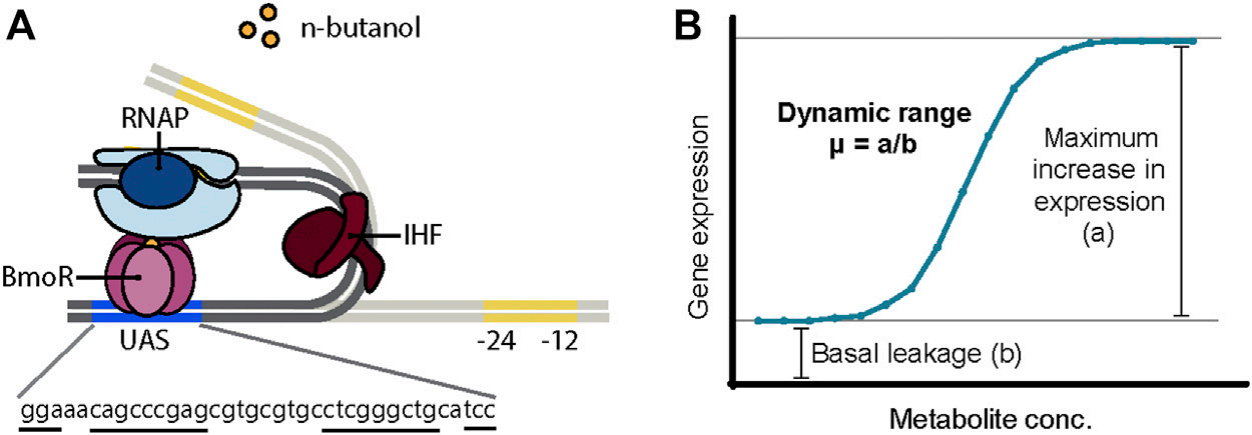
**(A)** Proposed mechanism of BmoR-P_BMO_-based biosensor. σ^54^-dependent promoter, P_BMO_, is regulated by alcohol-responsive bacterial enhancer protein (bEBP), BmoR. In the presence of butanol, BmoR forms a multimer complex and binds to the upstream activating sequence (UAS) of the P_BMO_ located 193 bp upstream from the σ^54^-RNA polymerase binding sites, −24 and −12 boxes. Due to the distance between the σ^54^-RNA polymerase and BmoR, long-distance regulation is achieved through DNA looping induced by the integration host factor (IHF) protein. **(B)** Biosensor parameters. The dynamic range of the BmoR-P_BMO_-based biosensor is altered through the mutagenesis of the putative UAS sequence. The dynamic range is the fold-change of the maximum expression in the presence of the ligand over the basal leakage in the absence of the ligand (Mannan et al., 2017).

In such instances, the BmoR-P_BMO_-based biosensor was demonstrated in its native form exhibiting a linear detection range for 1-butanol between 0 and 40 mM (∼3 g/L or ∼0.3%) for *n*-butanol (Dietrich et al., 2013) and isobutanol (Yu et al., 2019b) and a >10-fold linear dynamic range high enough to discriminate with a ∼700 μM 1-butanol concentration difference (Dietrich et al., 2013). However, there are plenty of opportunities to optimize and further characterize the BmoR-P_BMO_-based biosensor to meet specifications for industrial use. Thus far, BmoR protein itself was engineered to have a wider detection range and specificity up to 100 mM *n*-butanol (Yu et al., 2019a). However, the P_BMO_ promoter remains relatively uncharacterized and optimizing its function by sequence modification has not been attempted. P_BMO_ is a σ^54^-dependent promoter regulated by the *n*-butanol-responsive TF BmoR. σ^54^-dependent promoters are distinct in its mechanism of activation, structure, and conserved core sequences from σ^70^-dependent promoters (Bush and Dixon, 2012). While most σ^70^-dependent promoters activate transcription upon binding of σ^70^ factor to the −10 and −35 boxes, σ^54^-dependent promoters require additional events for gene activation after the σ^54^ factor recognizes a different set of core sequences −12 and −24 boxes (Figure 1A). σ^54^-dependent TF, also known as bacterial enhancer binding protein (bEBP), binds as a hexamer to the upstream activating sequence (UAS) located further upstream of the core sequences. Due to the distance between the core sequences and the UAS, DNA looping facilitated often by a bending protein, integration host factor (IHF), or intrinsically bent DNA enables the interaction between the bEBP with the RNA polymerase (Freundlich et al., 1992). Transcription is finally activated following ATP hydrolysis. Furthermore, high-throughput screens on non–growth-related phenotypes and dynamic butanol-dependent regulation represent powerful metabolic engineering strategies that are largely unavailable to these efforts. This capability gap is due to a lack of TF–promoter pairs with user-defined controls. Therefore, we need to understand its engineering potential.

In this work, we explore how changes in DNA sequence impact promoter function of the BmoR-P_BMO_-based biosensor using sort-seq. Understanding how changes in DNA sequence alter promoter function can provide insights to better identify functional regions in a sequence to engineer promoters with desired properties (Figure 1B). Specifically, the effects of thousands of P_BMO_ sequence variants were measured to identify sites that are essential to the function of the biosensor and those that increase the sensor output. This paper provides an efficient, high-throughput approach in tuning the dynamic range of promoters not previously characterized.

## Results

### Function of the P_BMO_ Hairpin

Transcription factor (TF)–based biosensors regulate transcription based on specific interactions between the protein and the DNA operator sequences. The DNA binding domain (DBD) of TFs interact with its respective promoters by recognizing specific operator sequences that often form hairpin structures affected by having inverted repeat sequences (Stanton et al., 2014; Rohlhill et al., 2017; Kim et al., 2020). Base changes in these palindromic regions have been shown to alter the binding affinity of the protein resulting in changes in the promoter’s strength (Stanton et al., 2014; Rohlhill et al., 2017). Truncation assays of the P_BMO_ promoter identified an inverted repeat sequence upstream of the TSS (Supplementary Figure S1A) that is necessary for promoter activity in the presence of 1-butanol (Dietrich and Keasling, 2013) (Figure 1A). Therefore, we first examined the function of the inverted repeat sequence here known as the P_BMO_ hairpin.

**FIGURE 2:**
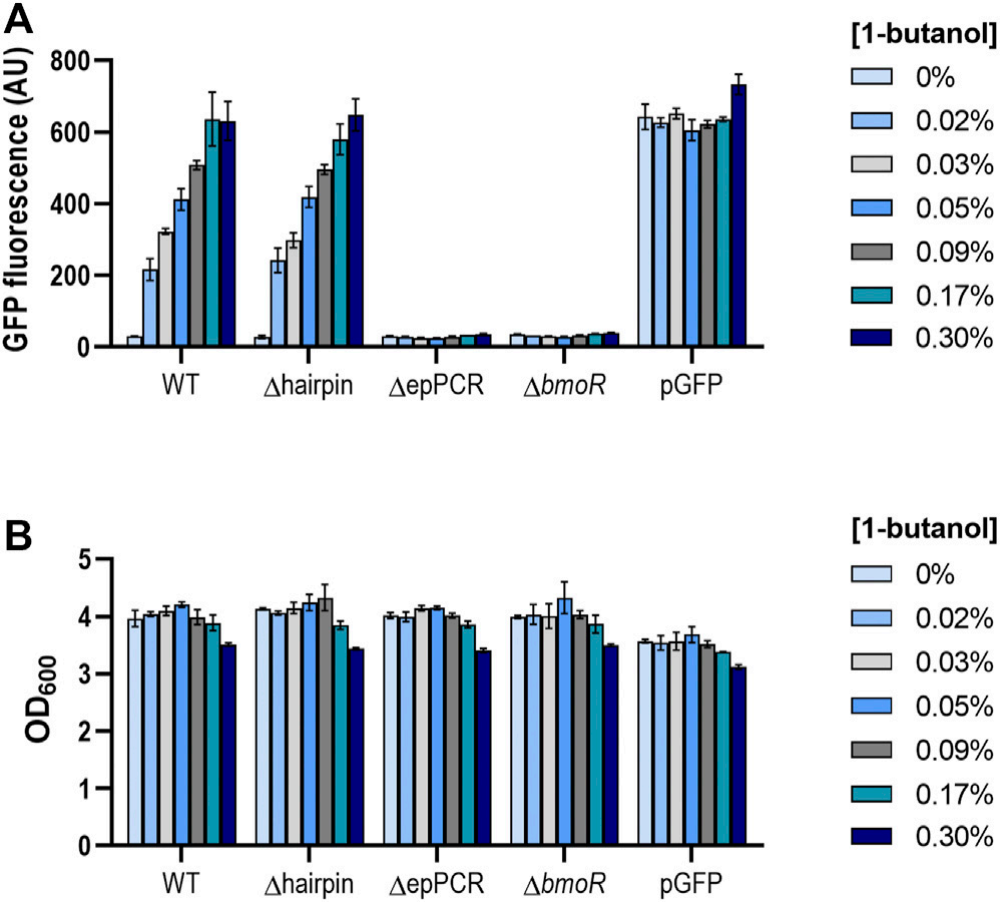
**(A)** P_BMO_ promoter without its hairpin feature elicits similar butanol response as the wild-type variant. The P_BMO_ promoter with (WT) and without the P_BMO_ hairpin (Δhairpin) were measured 16 h after 1-butanol induction (0, 0.02, 0.03, 0.05, 0.09, 0.17, 0.3% v/v) via flow cytometry. Removal of upstream promoter region (ΔepPCR) and plasmid without BmoR (ΔbmoR) were used as negative controls and pGFP, induced with 1 mM IPTG, as the positive control. Experiment was done in three replicates on a single day from three individual colonies (*n* = 3). Error bars represent the SD. **(B)** Butanol effects on cell growth. Cell densities (OD_600_) were also measured at the time of the GFP fluorescence measurements. Decrease in cell density was observed when induced greater than 0.3% 1-butanol (v/v).

To understand the role of the P_BMO_ hairpin in the context of the whole promoter, we compared the butanol response in the presence and absence of the P_BMO_ hairpin (Supplementary Figure S1B). Each promoter variant was cloned to control the expression of a *gfp* reporter in a vector carrying the *bmoR* gene under the control of its native constitutive promoter, P_BmoR_, resulting in WT and Δhairpin. Additional constructs were created without BmoR (Δ*bmoR*) and deletion of the upstream P_BMO_ sequences (ΔepPCR) to serve as negative controls. Promoter activities in *E. coli* NEB5α after *n*-butanol induction were measured via flow cytometry. When induced with high levels of butanol (0.53 and 0.6%), a rapid increase in fluorescence was observed along with lower cell density, which may be attributed to butanol toxicity, as seen in previous work (Supplementary Figure S2) (Dietrich et al., 2013; Yu et al., 2019a). Absence of BmoR resulted in no GFP expression when induced, indicating that no other endogenous proteins other than BmoR activate P_BMO_ (Figure 2A; Supplementary Figure S3). Interestingly, while deletion of the upstream sequences leads to complete inactivation of GFP, which is shown by construct ΔepPCR as previously shown (Dietrich et al., 2013), the Δhairpin showed a similar promoter activation profile as the wild-type promoter while exhibiting a low basal expression when induced with 1-butanol. These results demonstrated that the P_BMO_ hairpin alone may or may not be involved in BmoR binding.

### Sort-Seq on the P_BMO_ Promoter

**FIGURE 3:**
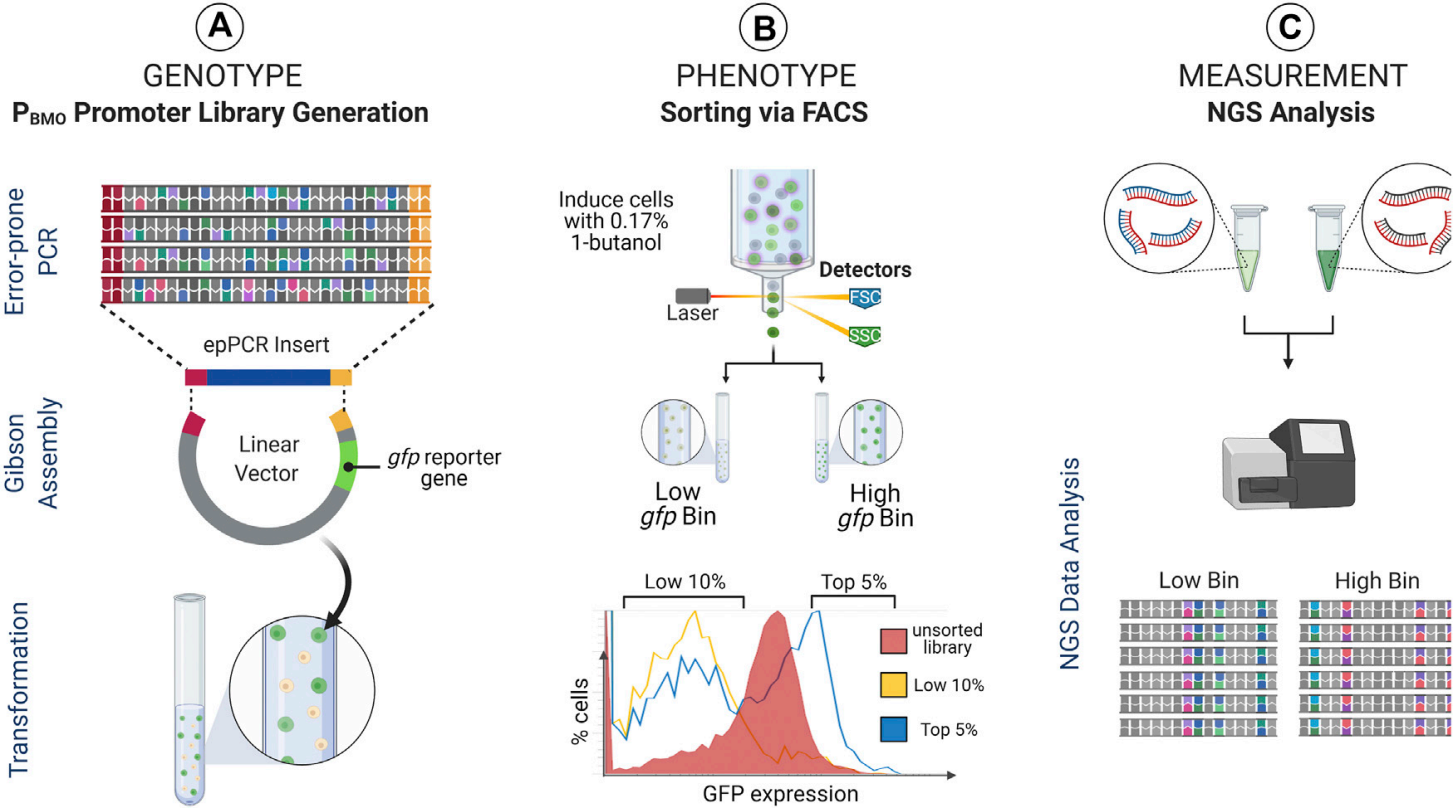
Sort-seq experimental strategy. Sort-seq enables measuring gene expression profiles of thousands of promoter library sequences pooled into a single pot. **(A)** A mutagenized P_BMO_ library (Genotype) is cloned upstream of *gfp* reporter gene in *E. coli* and **(B)** induced with butanol and sorted into activity-based (i.e., fluorescence-based) populations (Phenotype). **(C)** These populations are subsequently deep sequenced to identify P_BMO_ mutations that correlate with changes in *gfp* expression (Measurement). Identified P_BMO_ mutations are constructed and validated in *E. coli*, enabling construction of synthetic promoters with desirable characteristics.

Given that the removal of the putative BmoR binding site from the promoter elicited a regulatory response like the wild-type sequence, we expanded the investigation of the impact of the P_BMO_ hairpin and the surrounding sequence space of on BmoR binding interaction using sort-seq (Figure 3).

#### P_BMO_ Promoter Library Generation and Characterization

**FIGURE 4:**
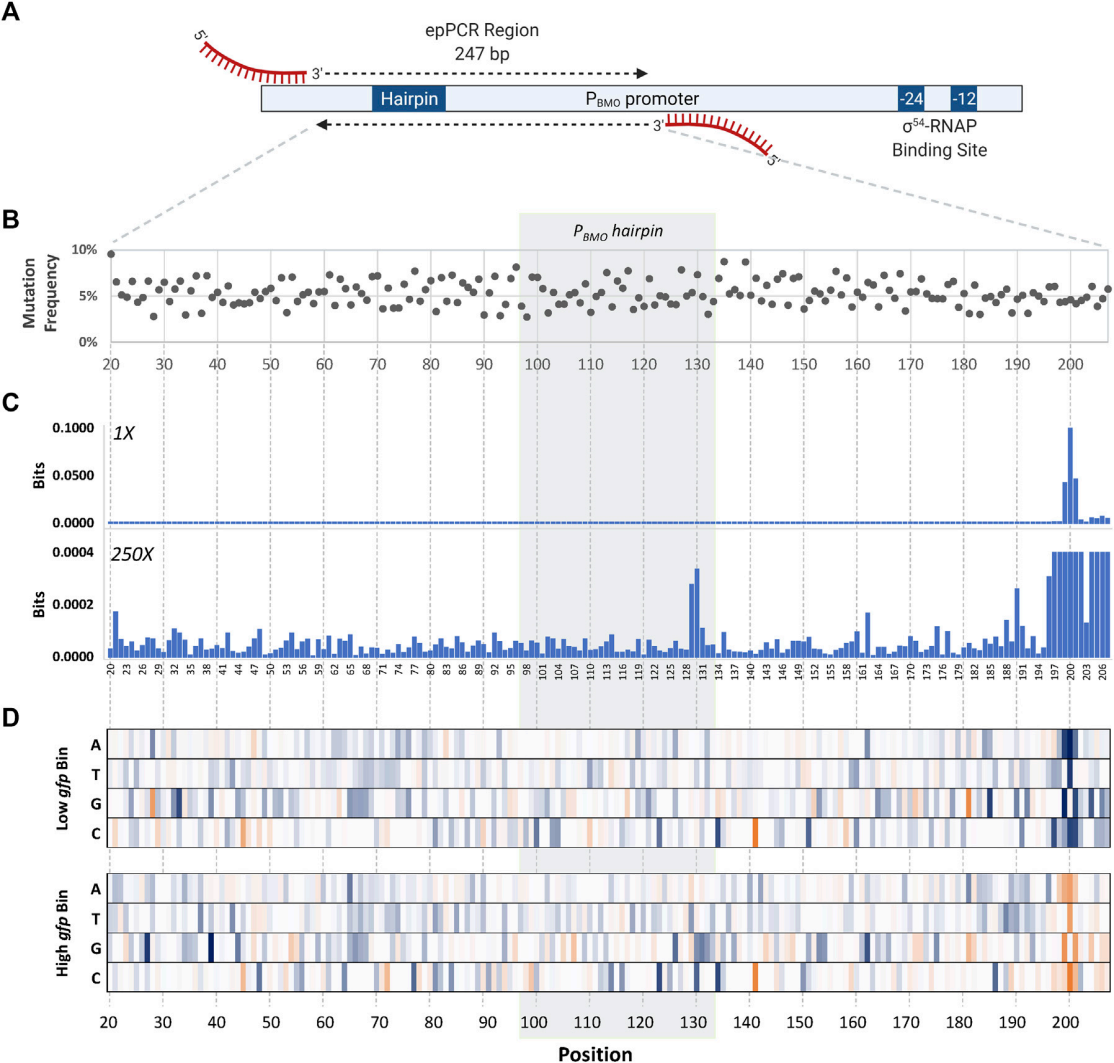
Identification of mutations that affect promoter strength using sort-seq. **(A)** Schematic of the mutagenized region in the wild-type P_BMO_ promoter. The length of the error-prone PCR region is 247 bp, located 112–359 bp upstream from the TSS (+1) in the P_BMO_ promoter. This region includes the putative BmoR binding site, P_BMO_. Deletion of this region inactivates promoter activity seen in Figure 2A. **(B)** The final P_BMO_ library resulted in an average mutation frequency of ∼5%. **(C)** Information footprints, generated using mutual information, identified sites that contribute to changes in *gfp* expression. Information footprint (1X) clearly identified sites critical in the proper function of σ^54^-based promoters, as well as the two mutation sites in the putative operator sequence (×250). **(D)** Enrichment heat maps of specific P_BMO_ sequences show details of enriched (blue) and rare or depleted sequences (orange) found at each position in each of the two sorting bins, low *gfp* and high *gfp*.

Determination of the mutagenized region was chosen based on previous data demonstrating complete loss in GFP expression in the P_BMO_ promoter truncates (Dietrich et al., 2013). The consensus region of the σ^54^ RNA polymerase binding site was not included since it is a well-known knowledge that mutations in this region negatively affect promoter activity (Figure 4A) (Buck et al., 2000).

The selected promoter region (Figure 4A) that includes the putative P_BMO_ hairpin, 247 bp in length, underwent five rounds of error-prone PCR (epPCR) to achieve the desired mutation of ∼5% at each nucleotide position. Preliminary assessment of mutation frequency was performed via Sanger sequencing of 10 members of the library. The epPCR-generated P_BMO_ library was inserted upstream of *gfp* using HiFi assembly. A total library size of ∼2.7 million P_BMO_ sequences was generated assuming that the library diversity and size are equivalent. Subsequent deep sequencing analysis confirms the high level of diversity of the unsorted library.

P_BMO_ promoter activities from the pooled P_BMO_ library was measured and sorted based on GFP fluorescence expressed 16 h post-induction with 0.17% butanol (v/v) (Figure 3B). Previous work using sort-seq found that variants sorted into the mid-level expression bins did not reveal any unique sequences that were different from the highest and the lowest expression bins (Rohlhill et al., 2017). Therefore, we sorted the induced library into two expression bins, the top 5% (high *gfp* bin) and the bottom 10% (low *gfp* bin). The P_BMO_ region of each bin was deep sequenced (Amplicon-EZ sequencing, GeneWiz) after amplification from extracted plasmids from ∼200,000 cells in low *gfp* bin and ∼500,000 cells in high *gfp* bin.

Sequences obtained from NGS were first pre-processed to filter out short and poor-quality reads prior to data analysis. Unique reads were aligned to the wild-type sequence and subsequently used to calculate and map the mutation frequency of the reads at each nucleotide position within the 247 bp length of the unsorted library (Figure 4B). The first and last 20 bases of the 247-bp region were removed from analysis due to the regions having a low mutation frequency due to the primer binding regions, shortening the analysis region to 207 bases in length. The final library contained ∼5% of the sequences mutated per position. Mutational bias analysis was also completed (Supplementary Table S1).

#### Identification of Position-Specific Sequences That Contribute to GFP Expression Levels

Mutual information scores were calculated to examine the effect of mutations between the fluorescence level and base at each nucleotide position in the region of interest as described in the methods. The mutual information of each nucleotide position generates an information footprint, which provides a visual map of nucleotide positions that governs gene expression and therefore provides potential targets for promoter engineering. In nucleotide positions where the base identity does not influence expression levels, the information content would be close to zero.

Information footprints identified positions inside and outside of the putative binding site to be important to the promoter function (Figure 4C). The information profiles portrayed a footprint that was clearly visible at three nucleotide positions, P199, P200, and P201, located 65 bp downstream of the P_BMO_ hairpin structure, overwhelming the values at other positions. A closer look in this region reveals that the surrounding regions P196–P207, except for P203, were sensitive to mutations with information bits ranging 3.03 × 10^−4^ to 9.99 × 10^−2^. Information values outside the P196–P207 region were in the low range of bits of 10^−6^ to 10^−4^. Although the information values are small in contrast to the P196–P207 region, there were two sites, P129 and P130, within the putative BmoR binding site observed to be more important than the flanking sequences.

Once we identified key nucleotide positions that affect gene expression using the information footprint analysis, we calculated the enrichment ratios between the distribution of mutations in the sorted library and the unsorted library for every variant to identify which base mutation at those identified sites altered gene expression (Figure 4D). A cluster of high information at sites P196–P207 were mostly mutations that correlated with low promoter function. Unfortunately, the P208–P247 region was not analyzed because of insufficient counts of mutations in this region due to its proximity to the epPCR primer binding region. Mutations other than the wild-type sequence in the P196–P207 region were highly enriched in the low bin heat map and mostly depleted in the high bin. Enrichment of positions P198–P201 profiled a higher conservation for the wild-type sequence in which sequences other than the wild-type sequence TGT at P199–P201 are severely depleted in the high bin while enriched in the low bin. These data indicate that if this region were mutated, it would lead to a loss in P_BMO_ function. The level of enrichment flanking this region are lower in the low bin but was still significantly higher in comparison with the sequences upstream of P196.

At the P_BMO_ hairpin site, an enrichment for base “T” and “C” were shown for P129 and P130, respectively, in the high expression bin and all four bases equally distributed in the low expression bin. These two sites are in the stem of the hairpin structure with no complementary sequence (Figure 7A).

#### Validation *via* Construction of Individual Mutants

To validate our findings, individual mutations were constructed and tested. We primarily focused on mutations that increased the dynamic range over a range of mutual information (MI) scores. Single sites identified as important in the information footprint and were enriched in the high *gfp* bin included nucleotide positions P129, P130, P162, P196, and P205. P200 was included to demonstrate a negative mutation. We also selected positions, P52, P77, P131, P133, P135, P185, and P188, with low MI values but had mutations enriched in the high *gfp* bin but depleted in the low bin as additional negative controls. A total of 12 single sites were selected.

**FIGURE 5:**
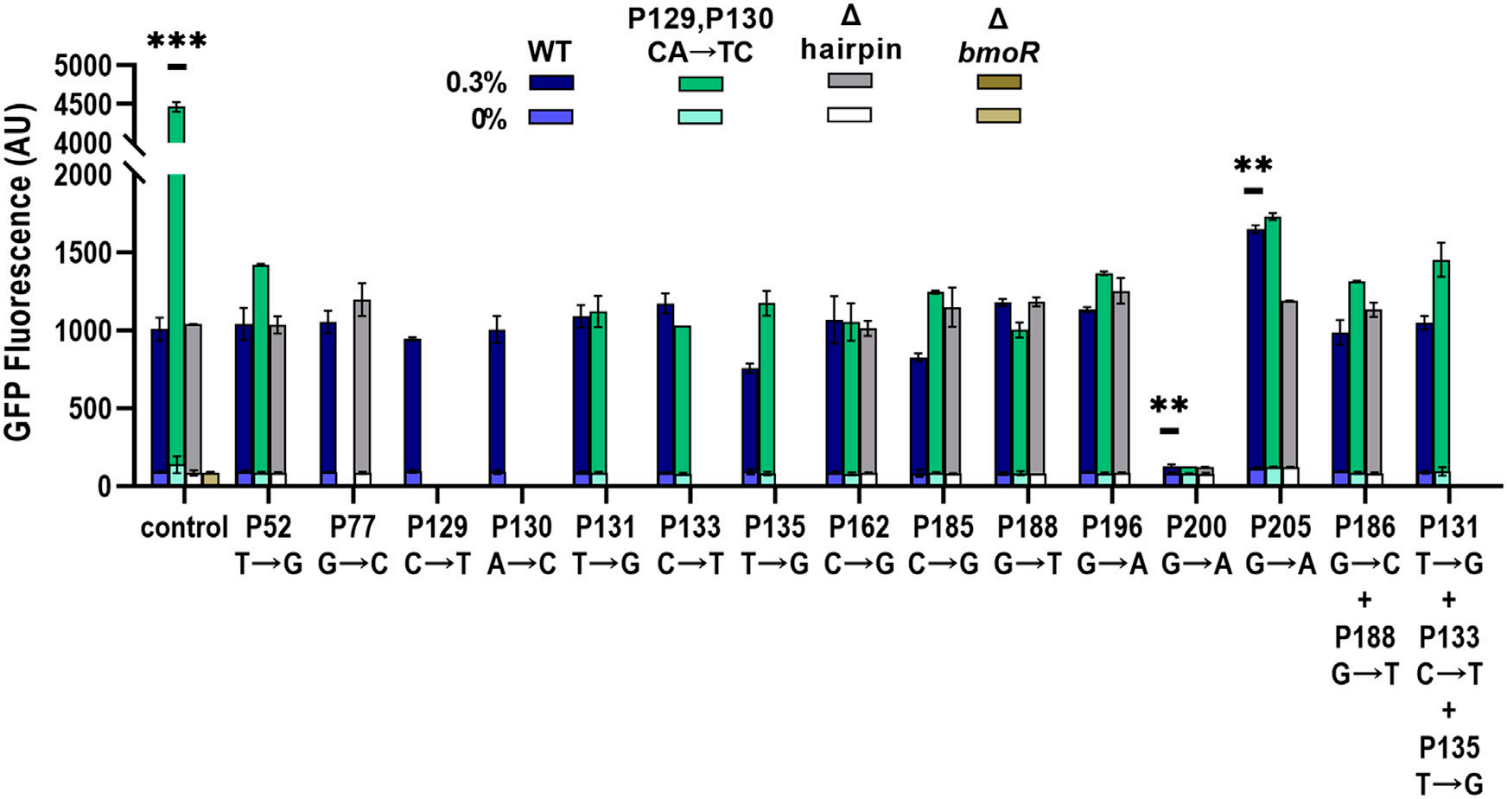
Validation of individual promoters with identified sites from sort-seq. Promoters were induced with 0 and 0.3% 1-butanol (v/v) and the *gfp* expression was measured by flow cytometry. Single and combinations of mutations were chosen based on the P_BMO_ hairpin architecture, wild-type (WT) (blue bars), CA(129,130)TC (green bars), and Δhairpin (gray bars), which is labeled in the x-axis as “control.” ΔbmoR was used as a negative control. Three promoters were found to alter the dynamic range, CA(129,130)TC, P200, and P205. Statistical difference of the 0.3% 1-butanol (v/v) induced samples between WT and variants were determined by a two-tailed *t*-test: CA(129,130)TC, *p* = 0.0004; P200, *p* = 0.0036; P205, *p* = 0.0074. Error bars show the SD (*n* = 2); *p*-value summary: ***p < 0.0001, **p < 0.005.

We were also interested in the additive effects of combinations of mutations based on the P_BMO_ hairpin. A total of 39 P_BMO_ variants with single or combination mutations were constructed into the P_BMO_ promoter carrying the wild-type hairpin (WT), Δhairpin, and combined double mutant hairpin variant CA(129,130)TC for validation. Validation of individual sequences were characterized by flow cytometry with 0 and 0.3% 1-butanol (v/v) (Figure 5).

Both P162 and P196 mutations as well as the negative controls, P52, P77, P131, P133, P135, P185, and P188, did not increase the dynamic range compared with the wild-type promoter with the different P_BMO_ hairpin variants. The two mutations in the P_BMO_ hairpin, P129 (C→T) and P130 (A→C), as single mutations did not affect expression. However, combining both mutations at P129 and P130 (CA→TC) increased its dynamic range by over 4-fold compared with the wild-type hairpin when induced with 0.3% 1-butanol (v/v). Mutations at P200 completely abolished biosensor function regardless of the type of hairpin variant. Although mutations within the P197–P207 region were mostly depleted in the high expression bin, P205 (G→A) was enriched in the high expression bin and had a large MI score. Incorporation of P205 (G→A) improved the promoter with the wild-type hairpin by 1.64-fold. However, combining G(205)A mutation with the CA(129,130)TC variant returned the level of promoter activation like the wild-type hairpin with the G(205)A mutation. Similarly, addition of other mutations to the CA(129,130)TC variant significantly reduced the promoter’s strength, while no effect was observed in the wild-type and the Δhairpin variant. Overall, no other single and combinations of mutants improved the dynamic range as the CA(129,130)TC variant.

### Further Investigation of CA(129,130)TC and G(205)A Variants Tuning Properties

#### Investigating CA(129,130)TC Promoter Properties

**FIGURE 6:**
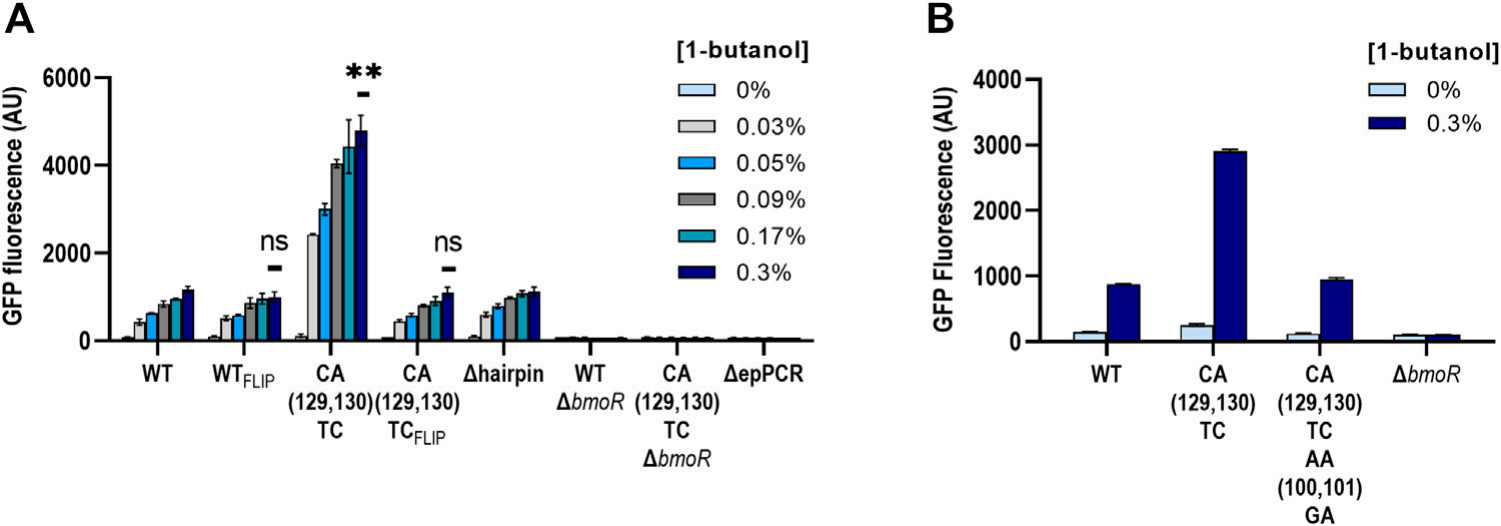
Biosensor properties of the CA(129,130)TC hairpin. **(A)** P_BMO_ hairpins of the wild-type (WT) and the CA(129,130)TC were flipped to investigate the modularity of the secondary structure. Both of the flipped hairpin structures, WT_FLIP_ and the CA(129,130)TC_FLIP_, were found to be none significant compared to the wild type. Statistical difference of the 0.3% 1-butanol (v/v) induced samples between WT and variants were determined by a two-tailed *t*-test: WT_FLIP_, *p* = 0.2344; CA(129,130)TC_FLIP_, *p* = 0.5645; CA(129,130)TC, *p* = 0.0048. Error bars show SD (*n* = 2). *p*-value summary: ***p < 0.0001, **p < 0.005, n.s. *p* > 0.05. **(B)** The CA(129,130)TC hairpin also did not maintain its high dynamic range when the sequences directly opposite were mutated to complement TC at P129 and P130.

The high activation level of the CA(129,130)TC variant was unique compared with other mutants; therefore, we further explored other properties of this mutant. To ensure that there were no other mutations in the plasmid causing the high dynamic range, we extracted the plasmid DNA from the samples ran and sequenced the whole plasmid of both with the wild-type and the CA(129,130)TC hairpin twice. No additional mutations or differences other than the mutations at the P129 and P130 sites were found. In addition, we retransformed the sequence-confirmed plasmids and retested the activity in fresh cells (Figure 6A). In the same experiment, we also investigated whether CA(129,130)TC P_BMO_ mutant alone is involved in the binding of BmoR in the presence of butanol. We constructed P_BMO_ promoter carrying the reverse complement of the wild-type (WT_FLIP_) and the CA(129,130)TC (CA(129,130)TC_FLIP_) hairpin sequence. If replacing the hairpin sequence by its reverse complement does not affect gene expression, then BmoR may be specifically binding to the CA(129,130)TC variant. We also included the CA(129,130)TC variant plasmid without BmoR (CA(129,130)TCΔ*bmoR*) to ensure that the effect was specific to BmoR. Replacing the P_BMO_ CA(129,130)TC hairpin with its reverse complement returned its expression strength to the same level as the wild-type hairpin (Figure 6A). Likewise, mutating the complementary side of the CA(129,130)TC to be symmetrical, shown by AACA(P100, P101, P129,P130)GATC, returned the dynamic range similar to the wild-type hairpin promoter strength (Figure 6B). The 4-fold higher expression compared with the wild-type exhibited by the CA(129,130)TC variant was reproducible after extraction and retransformation of the plasmid. BmoR appears to be the sole activator of the CA(129,130)TC promoter in the presence of butanol.

#### Altering the Dynamic Range Through the P_BMO_ Hairpin

**FIGURE 7:**
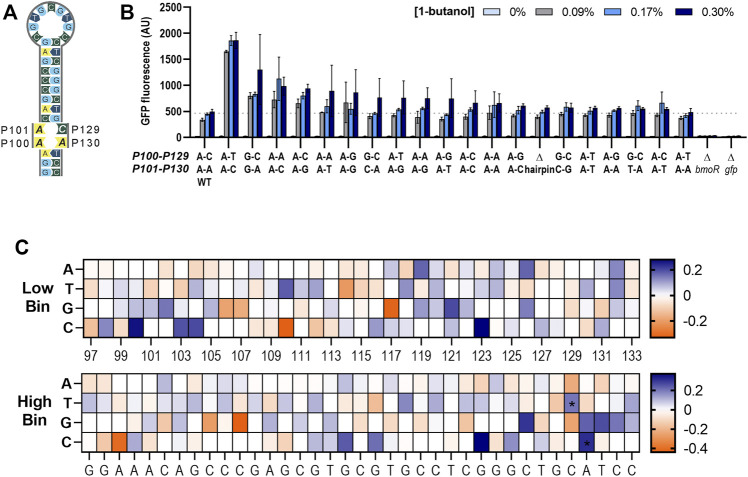
Altering the dynamic range through the P_BMO_ hairpin. **(A)** Schematic of the putative P_BMO_ hairpin mutants tested in this study. Putative P_BMO_ hairpin is a near-perfect palindromic sequence containing two nucleotide base pairing mismatches. **(B)** Activation strength of 19 P_BMO_ hairpin mutants are plotted based on the 0.3% 1-butanol (v/v) including constructs without *bmoR* (Δ*bmoR*), *gfp* (Δ*gfp*), and hairpin (Δhairpin) as controls. Each mutant was induced with 0, 0.09, 0.17, and 0.3% 1-butanol (v/v). Experiments were run in three replicates on three separate days from three individual colonies (*n* = 3). Error bars represent the SD. **(C)** Enrichment heat maps of the P_BMO_ hairpin region in P97–P133 show enriched (blue) and rare or depleted sequences (orange) found at each position in the low and high bins. Two positions at P129 and P130 found to contain high information in the information footprints were enriched with T at P129 and C at P130 annotated with symbol * in the high *gfp* bin.

P_BMO_ mutant carrying CA(129,130)TC in the hairpin sequence demonstrated that small base changes result in large effects on gene expression. This led us to further explore other nucleotide mutations at the P129 and P130 sites to alter the dynamic ranges of the butanol biosensor. A suite of 15 P_BMO_ promoters, not including the wild-type hairpin sequence, covering all possible mutant combinations at these two mismatched sites, P129 and P130, on the right half-site of the hairpin sequence were constructed (Figure 7A). Four additional mutants with mutations at P100 and P101 were added to create a range of binding strengths, resulting in a total of 19 P_BMO_ hairpin mutants and the wild type. Evaluation of the promoters containing the hairpin mutants demonstrated that the CA(129,130)TC variant significantly outperformed the others (Figure 7B). None of the mutations performed below the wild-type promoter, which is consistent with the enrichment data and Δhairpin studies. A two-way ANOVA of the 0.3% 1-butanol induced samples agree that the strength of the *gfp* activation is determined in a site-specific sequence preference for nucleotide “T” at P129, while site P130 had a slightly more dependence on the DNA sequence for “C” (Supplementary Figures S4A–F) and is consistent with the enrichment data (Figure 7C).

#### Tuning the Dynamic Range via the Integration Host Factor Region

**FIGURE 8:**
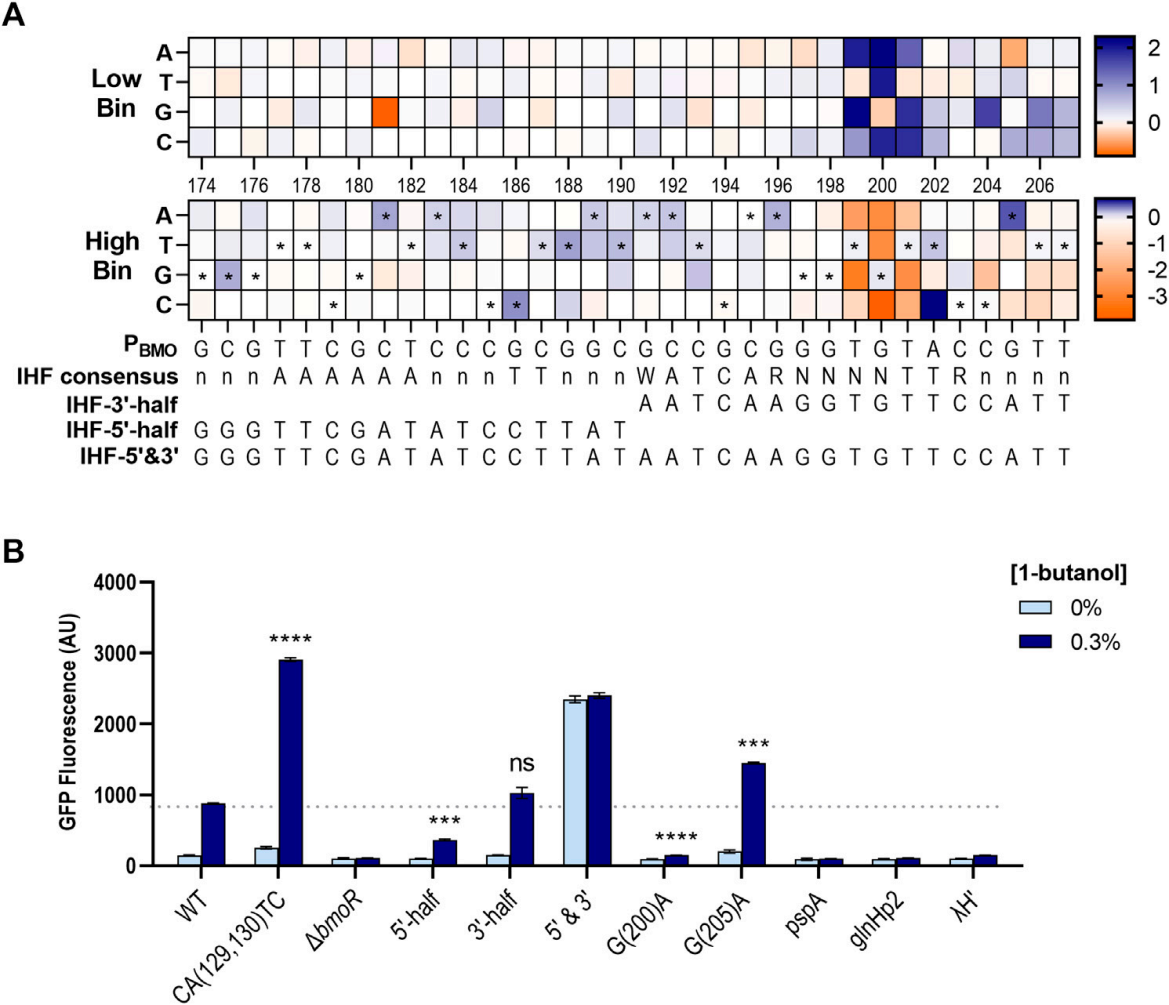
Enrichment maps exposes preferred sequence of the P_BMO_ promoter in *E. coli*. **(A)** Enrichment map of P174–P207 region shows enrichment for the putative *E. coli* IHF binding sequence, which is annotated by the symbol * in the high *gfp* bin. The *E. coli* IHF consensus sequence is listed below the heat maps, along with the mapped sequences. Enriched (blue) and rare or depleted sequences (orange) found at each position in the low and high bins. **(B)** Three different promoters with different forms of the putative IHF sequences were constructed based on the enriched sequences from the heat maps **(A)**: P174–P190 (IHF-5′-half), P191–P207 (IHF-3′-half), and P174–P207 (IHF-5′&3′). Three additional constructs with IHF sequences from three known σ^54^-dependent promoters, *pspA*, *glnHp2*, and λH′. Statistical difference of the 0.3% 1-butanol (v/v) induced samples between WT and variants were determined by a two-tailed *t*-test: 5′-half, *p* = 0.0005; 3′-half, *p* = 0.1155; G(200)A, *p* < 0.0001; G(205)A, *p* = 0.0004. Error bars show SD (*n* = 2); *p*-value summary: ***p ≤ 0.0001, n.s. *p* > 0.05.

Mutual information scores identified the P196–P207 region as highly important in promoter function. We mapped the non-depleted sequences in the region and near surroundings and found the P191–P207 region to have a sequence homology with the consensus sequence of the *E. coli* Integration Host Factor (IHF) binding site (Aeling et al., 2006; Figure 8A). IHF does not directly affect transcription, but is required in facilitating optimal protein–protein interactions between the TF and RNA polymerase (Freundlich et al., 1992). IHF binds in a sequence-specific manner contacting ∼34 bp (5′-nnnAAAAAAnnnTTnnnWATCARnnnnTTRnnnn-3′) (where W = A/T, R = A/G, and *n* = any base) (Aeling et al., 2006), where the 3′-half of the binding site are conserved with the sequence WATCARnnnnTTR across *E. coli* (Freundlich et al., 1992). Mutations consistent with the WATCAR motif is shown to be enriched at P191–P196 in the high expression bin, where the wild-type sequence is GCCGCG. The TTR element was not present at P201–P203 as expected by the consensus motif, but rather found further upstream at P181–P190 where it forms a palindrome with the sequences at P191–P200. This was not found to affect biosensor function in our mutual information or enrichment data.

Although most of the sequences identified in the IHF region were mutations that suppressed promoter function, a few mutations were enriched in the high *gfp* bin (Figure 8A). Thus, we investigated if it was possible to tune the dynamic range through the putative IHF binding site. We created three new constructs where only the 5′-side of this region were mutated with the enriched sequences found in P174–P190 (called “IHF-5′-half”), only the 3′-side were mutated with enriched sequences in P191–P207 (“IHF-3′-half”), and both sides are mutated (“IHF-5′&3′”) (Figure 8A). In addition, we included IHF sites from three known σ^54^-dependent promoters, P_pspA_, P_glnHp2_, and P_λH_′ (Aeling et al., 2006).

Overall, the IHF binding site was not easily as tunable as the single P205 mutant (Figure 8B). Replacing the 3′-half with the identified enriched sequences, which contains a partial IHF consensus sequence, had no effect on the dynamic range shown by the construct IHF-3′-half. However, mutating the 5′-half with the enriched sequences reduced the dynamic range by ∼3-fold compared with the wild type. Interestingly, mutating both sides of this region increased GFP expression in the presence of 0.3% 1-butanol, but the basal expression significantly increased in the absence of butanol. The IHF-5′&3′ mutant promoter appeared to be in an active state without the help of BmoR. Replacing the original sequence with other IHF sites from P_pspA_, P_glnHp2_, and P_λH_′ completely eliminated expression.

## Discussion

The success of creating a suite of promoters with different strengths would enable user-defined promoters to be of high value to commercial and industrial applications. In this work, we engineered a butanol responsive transcription factor (TF)–promoter to increase its dynamic range through sequence–function elucidation of the promoter region that interacts with the TF with sort-seq. The effects of >10^6^ DNA sequence variants were measured based on the level of *gfp* expression activities from a mutagenized library to develop a predictive model of gene regulation gaining insights into P_BMO_ promoter function. Through single-base pair modeling, two promoter variants were identified that exhibited significant improvements in the dynamic range with increased sensitivity for its analyte, while keeping low basal levels. Combining double mutant CA(129,130)TC demonstrated a 4-fold improvement, while single mutant at P205 performed 1.64-fold greater in the dynamic range over the native promoter sequence.

Sort-seq was especially useful in predicting key binding sites of uncharacterized promoter architectures. Prior to performing sort-seq, an initial assessment of the P_BMO_ promoter found that BmoR was able to activate *gfp* expression without the hairpin sequence at the same expression level as the wild type. Other bacterial enhancer binding proteins (bEBPs) have been shown to activate their respective promoters with deleted UAS sites, but activate at a weaker state than promoters with the UAS sites (Claverie-Martin and Magasanik 1991; Dworkin et al., 1997). In these cases, higher levels of bEBPs were required to activate equivalent transcription (Dworkin et al., 1997). Few studies have shown that bEBP NtrC is capable of forming stable complexes on non-specific DNA through superhelical regions of supercoiled plasmid templates that carry the promoter lacking the UAS site (Popham et al., 1989), even if there is no sequence homology to the UAS (Brahms et al., 1995). Despite wild-type-like activation of the deleted hairpin promoter variant, altering the dynamic range was still possible through the P_BMO_ hairpin shown by the CA(129,130)TC promoter. Information footprints revealed two nucleotides within the P_BMO_ hairpin with high mutual information. Also, identification of the P205 variant would have not been predicted if the P_BMO_ hairpin sequence was the sole focus on regulation.

Factors such as flanking DNA sequences of operator sequences, DNA shape, and other cofactors in the host can affect the interactions between the promoter and the activating protein (Slattery et al., 2011). Sort-seq has been used to find TFs that activate promoters with other proteins (Kinney et al., 2010; Barnes et al., 2019). P_BMO_ is a σ^54^-dependent promoter regulated by BmoR. Enrichment analysis in the P196–P207 region of the high *gfp* bin identified sites of what could be the binding site of the *E. coli* IHF heterodimer protein, containing the IHFα and IHFβ subunits, which is required for the proper function of most σ^54^-dependent promoters in *E. coli*. In most cases, σ^54^-dependent promoters contain a binding site for the DNA bending protein, IHF, to facilitate DNA bending for transcription activation. Crystal structures of the IHF–DNA complex suggest that the DNA conformation formed by the WATCAR sequence is favored by the arm of the IHFα subunit (Rice et al., 1996). While the IHF binds to the WATCAR region through water-mediated hydrogen bonds, IHF directly interacts with only the TTR element (Rice et al., 1996). In the P_BMO_ promoter, sequences corresponding to the WATCAR motif were enriched in the high *gfp* population in P191–P196, but TGT was found instead of the TTR motif in P199–P201. Although P_BMO_ had TGT sequence, mutating G→A at P200 completely abolished P_BMO_ activity for all three promoter hairpin variants in a similar way where mutating the center nucleotide in the TTR element from T→A severely weakens the IHF–DNA complex (Lee et al., 1991; Lynch et al., 2003). Furthermore, promoter activation was also maintained similarly to the native P_BMO_ promoter sequence when this region was replaced with the *E. coli* IHF binding site which was shown by IHF-3′-half (Figure 6). However, the presence of an IHF binding sequence in the promoter based on enrichment analysis is insufficient. Although P_BMO_ is native to *Thauera butanivorans* and did not contain the *E. coli* IHF binding site consensus sequence, activation in *E. coli* was successful in its native sequence. It is possible that the P_BMO_ promoter might have an intrinsically bent region which is present in some promoters without IHF binding sequence (Goodman et al., 1999).

Due to the diversity and the number of σ^70^-dependent promoters, many promoter studies have been performed on σ^70^-based promoters. σ^54^-dependent promoters regulate expression in a variety of mechanisms that are yet to be uncovered. Elusive understanding of σ^54^-dependent promoters has hindered the use of σ^54^-dependent promoters in building genetic parts for synthetic biology applications. The fact that we were unable to explain the mechanisms of the BmoR-P_BMO_-based biosensor presented some limitations in using the sort-seq method. First, although the sort-seq method and the mutual information statistics were capable of identifying sites important in P_BMO_ transcription, it was difficult to determine whether BmoR acted alone or cooperatively with other factors (Barnes et al., 2019). Promoters with little knowledge about its regulation have used sort-seq with DNA affinity purification and mass spectrometry to identify proteins and their binding sites as well as their regulatory functions (Belliveau et al., 2018). Further studies specific to BmoR binding to its sequence motif could provide insights into its modularity. This would enable designing of new hybrid butanol-responsive genetic circuits.

Second, sort-seq does not appear to identify secondary structures, largely due to the practical limitation of the library size and subsequent number of sequencing reads obtained. P_BMO_ consists of multiple secondary structures, in addition to the P_BMO_ hairpin, throughout the promoter. This study would have benefited by performing an initial secondary structure analysis of the whole promoter in determining the region of interest. In some σ^54^-dependent promoters, oligomerization of bEBPs is induced by the DNA structure of the UAS sites. As the bEBPs bind to the UAS sites, the increase in the local concentration stimulates the formation of multimer complex and subsequently transcriptional activation by σ^54^-RNAP (Porter et al., 1993). The binding of bEBP causes the DNA to bend and wrap around the oligomerized protein (De Carlo et al., 2006). NorR from *E. coli* is dependent on the presence of all three binding sites to properly oligomerize for the activation of the *norV* promoter (Tucker et al., 2010). For CelR from *C. acetobutylicum*, introducing mutations in only one of three UAS sites severely diminished activity (Nie et al., 2016). NtrC binds to two binding sites in the promoter but recruits a third dimer to form a hexamer when the sensing domain is phosphorylated (De Carlo et al., 2006). Alternatively, σ^54^-bEBP regulators exist as dimers but assemble into a hexamer form when the inducer is bound or phosphorylated by a histidine kinase in the N-terminal domain (Bush and Dixon, 2012). BmoR consists of a butanol sensing domain in the N-terminal domain, the central domain to interact with the σ^54^ RNA polymerase, and the carboxyl-terminal domain to bind to the UAS of the P_BMO_ promoter. However, alignment analysis of the amino acid sequence between BmoR and other similar σ^54^-bEBPs showed that BmoR lacks the phosphorylation residue in the N terminus (Dietrich et al., 2013). Therefore, we initially assumed that BmoR existed as dimers but then formed a hexamer in the presence of 1-butanol. Including a sort-seq experiment under uninduced conditions could be helpful in determining the binding site preferences of BmoR as a dimer compared with the binding site preferences of heterodimerized BmoR when induced.

Lastly, this work was completed in the model organism *E. coli*. While the σ^54^-dependent BmoR-P_BMO_-based biosensor from *T. butanivorans* was compatible in *E. coli*, compatibility of the biosensor in other biobutanol-producing microorganism platforms needs to be investigated to realize the full potential of this tool.

Although we were unable to provide a proposed mechanism for how the σ^54^-dependent system, BmoR-P_BMO_-based biosensor, activates gene expression, we identified and functionally characterized important sites at the nucleotide level to increase the dynamic range in *E. coli*. This work can potentially enable high-throughput strategies to edit biosensor parameters of industrially relevant TF-based biosensors.

## Materials and Methods

### General

All *in vivo* experiments were performed within the NEB5α strain (New England BioLabs). Plasmids used in the study are described in Supplementary Table S2. Sanger sequencing and NGS were performed by GENEWIZ from Azenta Life Sciences (New Jersey). All primers used in this study were synthesized by Integrated DNA Technologies (IDT) and are listed in Supplementary Tables S3, S4.

### Plasmid Construction of P_BMO_ Hairpin Mutants, Sort-Seq Identified Promoter Validations, and Controls

Mutations, insertions, and deletions were incorporated or removed via PCR using Q5 High-Fidelity 2X Master Mix according to the manufacturer’s instructions (New England BioLabs) and primers listed in Supplementary Table S2 with the pNK25 plasmid as the starting template in 10-μl volume reactions. PCR products were visualized on 0.8% agarose to confirm size and successful amplification and subsequently purified using Qiagen QIAquick PCR Purification Kit. The plasmid template was removed using DpnI (New England BioLabs), then the 3′ ends of the PCR products were phosphorylated using T4 Polynucleotide Kinase (New England BioLabs) and ligated with Instant Sticky-end Ligase Master Mix (New England BioLabs) prior to transformation into chemically competent NEB5α (New England BioLabs). Transformants were selected on LB agar containing 100 μg/ml ampicillin (Amp^100^). Colonies were picked for overnight cultures for plasmid miniprep and then sent for Sanger sequencing.

### P_BMO_ Promoter Analysis *via* Flow Cytometry

Biosensor plasmids in *E. coli* NEB5α strains were cultured overnight in Luria–Bertani (LB) medium (250 rpm, 32°C; Thermo MaxQ) supplemented with 100 μg/ml ampicillin (Amp^100^). Overnight cultures were inoculated to an optical density at 600 nm (OD_600_) of 0.05 in 3 ml of fresh LB with Amp^100^ medium and grown to an OD_600_ of ∼0.20 (250 rpm, 32°C; Thermo MaxQ). Cultures were diluted in LB with Amp^100^ medium with or without 4X 1-butanol (3:1) to a final volume of 500 μl in 5-ml culture tubes and incubated for a total of 16 h (250 rpm, 32°C; Thermo MaxQ). End-point measurements of cell growth (OD_600_, DeNovix Ds-11+) and GFP fluorescence (Attune NxT flow cytometer; ThermoFisher) were measured. Cells were diluted 1:10 in the culture medium to measure the absorbance at 600 nm in 45 mm × 10 mm × 10 mm (H × W × D) cuvettes (Greiner). For flow cytometry GFP fluorescence measurements, 5 μl of cells were diluted in 500 μl of phosphate buffered saline, pH 7.4 (ThermoFisher), and the geometric mean of 10,000 events per sample were measured. A blue solid-state laser (488 nm excitation), an optical filter at 530/30 nm for GFP fluorescence, and 488/10 nm optical filter for side scatter (SSC) were used. Flow Cytometry Standard (FCS) files were used to analyze using the Attune NxT Software (ThermoFisher). The geometric means of the FITC-A fluorescence (in arbitrary units, AU) were taken for 10,000 events per sample.

### Promoter Library Generation and Assembly

Five rounds of error-prone PCR (epPCR) were completed with primers with overhangs designed for HiFi assembly upstream sequences of P_BMO_ using the GeneMorph II Random Mutagenesis Kit (Agilent). PCR products from each round of epPCR were purified using Qiagen QIAquick PCR Purification Kit and used as the starting template to obtain higher mutation rates. The first epPCR round used a starting template concentration of 10 ng of the 267-bp target region of the 5,788-bp-sized pNK25 plasmid using the manufacturer’s specifications. The plasmid template was removed with DpnI enzyme before the next round of epPCR. The second and the third epPCR rounds started with 0.1 ng of the PCR product from the previous epPCR round as the starting template. EpPCR rounds four to six used 0.01 ng of the PCR products from the previous round to increase the mutation rate.

The library vector, which includes all parts of the pNK25 plasmid but the 267-bp mutagenized region of interest in the P_BMO_ promoter, was amplified from 0.4 ng of pNK25 plasmid backbone as described previously, and the resulting PCR vector fragment was purified and treated with restriction enzyme DpnI to remove the wild-type plasmid template. To further mitigate the presence of the wild-type promoter in the final library, another round of PCR of the vector was done using 0.04 ng of the purified vector fragment from the first round of PCR in a 50-μl PCR reaction volume. The double-reverse PCR protocol resulted in no colonies after bacterial transformation confirming no template contamination in the vector DNA.

To gauge the mutation rate (mutations/kb) of the different rounds of epPCR, rounds 3, 5, and 6 of epPCR products were inserted into the pNK25 vector and transformed into *E. coli* prior to building the promoter library at a large scale. For each round, 10 individual colonies were picked from the transformation plates for colony PCR of the library insert, purified, and then sent for Sanger sequencing.

The full-scale R5 epPCR P_BMO_ promoter library was inserted into the vector using NEBuilder HiFi DNA Assembly Master Mix (New England BioLabs). All 20 μl of HiFi assembly reaction were electroporated (1-mm gap cuvettes; pulsed at 1,800 V; BioRad MicroPulser) into 10 aliquots of 50 μl of electrocompetent NEB5α cells. The cells were recovered in 250 μl of SOC media (New England BioLabs) per transformation. A small sample of the library was plated on solid LB Amp^100^ medium after the 1-h recovery post-electroporation to calculate the approximate library size. After 1-h recovery in SOC, the transformed library was combined in a 250-ml baffled flask containing 30 ml of LB Amp^100^ for antibiotic counterselection (250 rpm, 37°C; Thermo MaxQ), and sampled every half hour on the flow cytometer (Attune NxT, ThermoFisher) to monitor the population’s decay, plateau, and recovery using the FSC and SSC dot plot. Once the measured viable cell count of the untransformed cells had decreased and the cells harboring the promoter libraries began to increase (∼3 h), the library culture was immediately cooled on ice to maintain the library diversity. Cultures with the promoter library were aliquoted in 2-ml cryovials with sterile 20% (v/v) glycerol and stored at −80°C.

### Library Sorting

The library seed cultures were started from 2 to 4 frozen glycerol library stocks inoculated into 50 ml LB Amp^100^ and grown in a 250-ml baffled flask (200 rpm, 32°C). When the cultures reached an OD_600_ of ∼0.20 (8–9 h), the library was induced with 0% or 0.17% 1-butanol (v/v) and incubated for 16 h (250 rpm, 30°C; Thermo MaxQ). Library cultures were normalized to an OD_600_ of ∼0.1, further diluted by a factor of 4 in phosphate buffered saline, pH 7.4 (ThermoFisher), and placed on ice prior to sorting. Cells were sorted based on GFP expression into two bins at a rate of ∼9,000 events per second performed on the iSort Automated Cell Sorter with a blue solid-state laser (488 nm, 165 mW), optical filters 525/50BP for GFP and 488/10 SSC, 85 μm ceramic nozzle with a fixed sample flow rate of 23 μl/min (ThermoFisher). Low *gfp* bin contained the top 5% (∼200,000 events) and high *gfp* bin with the bottom 10% (∼50,000 events) of the population. A small amount of the sorted samples was measured on the Attune NxT flow cytometer to ensure successful separation and purity. Sorted samples were recovered in 10 ml LB Amp^100^ medium overnight (250 rpm, 32°C; Thermo MaxQ) and plasmid miniprepped (Qiagen QIA Spin Miniprep Kit) for NGS sample preparation. Concentration of the extracted plasmid DNA was measured at an absorbance at 600 nm (DeNovix Ds-11+).

### Deep Sequencing Sample Preparation

Plasmids from unsorted and sorted populations were extracted (Qiagen QIAprep Spin Miniprep Kit) and used as template for PCR. Sequencing libraries were amplified from 1,000 ng of the plasmid template with Q5 High-Fidelity 2X Master Mix for 10 cycles of PCR using primers with partial Illumina adapters sequence overhangs on the 5′ end (Supplementary Table S3). After confirming the expected amplicon size (312 bp) on a 0.8% agarose gel, amplicons were gel purified to remove the plasmid template and other PCR artifacts. The excised gel was purified using the Qiagen QIAquick Gel Extraction Kit with a modified protocol; the gel slice was dissolved in the QG buffer at room temperature while vortexing occasionally, the isopropanol was cooled to −20°C prior to adding to the QG buffer, and centrifuged at ∼4,500×*g* for 1 min per spin for the loading of the DNA. Subsequently, the gel purified amplicons were purified (Qiagen QIAquick PCR Purification Kit) to remove any additional impurities. The final concentration of the amplicons was measured at an absorbance at 260 nm (DeNovix Ds-11+) and normalized to 500 ng DNA in 25 μl of Tris–Cl, pH 8.0 with A260/280 at 1.8–2.0. Amplicons were sequenced using 2 × 250 bp paired end Amplicon EZ Sequencing service (GeneWiz).

### NGS Pre-Processing Workflow

Tulane University’s High-Performance Computer, Cypress, was used to analyze resulting FASTQ data. The raw sequence read count was found with a custom python script. Paired-end reads are interleaved and merged using BBmerge with default parameters (Bushnell et al., 2017). Merged FASTQ files were converted to FASTA format using BioPython (Cock et al., 2009). Using custom python scripts, sequences were filtered to remove those under 247 bp, the length of the reference sequence. Redundant reads were then collapsed. To remove hypermutated sequences, the hamming distance between each read and the native sequence was calculated and reads with a hamming distance greater than 20 were thrown out. The sequences were then aligned to the reference sequence and used to generate nucleotide counts per position using in-house custom Python scripts.

### Calculating Mutual Information for Generating Information Footprints and Enrichment Heat Maps

Information footprints and the enrichment heat maps were calculated as described in Rohlhill et al. (2017). In brief, the nucleotide occurrence of each base at each position were counted and normalized to the total number of reads in each library for the input library and the sorted high and low expression bin libraries. The mutual information between the expression bins and the bases at each nucleotide position were calculated using

$$I\left(b_{i};\mu \right)\approx \underset{b_{i},\;\mu }{\sum }f\left(b_{i},\mu \right)log_{2}\frac{f\left(b_{i},\mu \right)}{f\left(b_{i}\right)f\left(\mu \right)}$$

where $b_{i}$ is the base at the i th position, μ is the expression activity bin, and $f\left(b_{i},\mu \right)$ is the joint frequency distribution and $f\left(b_{i}\right)$ and f(μ) are the marginal frequency distributions. To calculate the enrichment of each base at each position, the log_2_ ratio of the number of nucleotide occurrences in each of the expression bins was normalized to the counts of the unsorted bins.

### Individual Promoter Validations

Biosensor plasmids in *E. coli* NEB5α strains were cultured overnight in LB medium (250 rpm, 32°C; Thermo MaxQ) supplemented with 100 μg/ml ampicillin (Amp^100^). Overnight cultures were inoculated to an optical density at 600 nm (OD_600_) of 0.05 in 3 ml of fresh LB with Amp^100^ medium and grown to an OD_600_ of ∼0.20 (250 rpm, 32°C; Thermo MaxQ). Cultures were diluted in LB with Amp^100^ medium with or without 4X 1-butanol (3:1) to a final volume of 160 μl in sterile Corning 96-well flat clear bottom black microplates and sealed with breathable rayon film (VWR). The OD_600_ of the 1-butanol induced cultures was measured every 15 min for 18 h in the plate reader (Molecular Devices SpectraMax iD5, shaking medium intensity, 30°C). End-point measurement of GFP was measured on the flow cytometer (Attune NxT; ThermoFisher) using the same flow cytometer parameters as described previously.

### Calculation of GFP Fluorescence Intensities and Cell Densities (OD_600_)

Flow cytometry histograms were generated with the Attune NxT acquisition software. Replicates of the geometric mean of the fluorescence of a population and the OD600 measurements for individual samples were measured and averaged in GraphPad Prism 9.2.0.332. The means with SDs were used to plot graphs.

### Visualization of the Information Footprints and Enrichment Maps

The information footprints and enrichment heat map calculations were completed in Microsoft Excel and then transferred to GraphPad Prism 9.2.0.332 to generate the plots.

## Data Availability

The datasets presented in this study can be found in online repositories. The names of the repository/repositories and accession number(s) can be found below: NCBI, PRJNA787372.
