# Supplementary material for: Elucidation of Sequence–Function Relationships for an Improved Biobutanol In Vivo Biosensor in E. coli
Source: Front Bioeng Biotechnol. 2022 Feb 21;10:821152. doi: 10.3389/fbioe.2022.821152 (PMC8899819; doi:10.3389/fbioe.2022.821152)
Supplement: Supplementary file 1 [file DataSheet1.PDF]

Supplementary Material for

Elucidation of sequence-function relationships for an improved biobutanol *in vivo* biosensor in *E. coli*

Nancy M. Kim[1], Riley W. Sinnott[2], Lily N. Rothschild[2], and Nicholas R. Sandoval[2]*
[1]Interdisciplinary Bioinnovation PhD Program, Tulane University, New Orleans, LA
[2]Department of Chemical & Biomolecular Engineering, Tulane University, New Orleans, LA
***Correspondence:**
Corresponding Author
nsandova@tulane.edu

Supplementary Figures

A

P_{BMO} Promoter Sequence

ccacagatagtaggtgctgcggctgctcatgctcctgtcgcggtagcgcgctgttacgcgaccgccccc
ggacctcggcggacagcgcggaagatt<u>ggaaacagcccgagcgtgcgtgcctcgggctgcatcc</u>ttgcc
acacccaaccggattcgtcggaccgctcgacattcgcgttcgctcccgcggcgccgcgggtgtaccgtt
gcgttacagatgtacccttctttaacgtgtaacacacgcctggagcggccaagagccccgcaccttgcg
gcgcgtcttccccaggggcccaccggttgcggccttttgctgcgaccgtccatgctggcacgacacttg
ctgaaagcgttagagcggaatcggtccgatggagcattcgaagccgctaccgacagcagaacacacaa

B

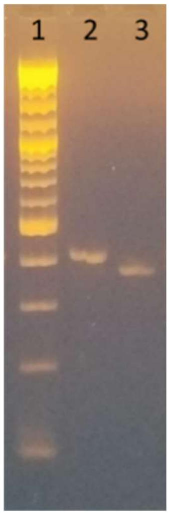

Supplementary Figure 1. (a) P_{BMO} promoter sequence with annotations (error-prone PCR region, putative operator, σ^{54} binding site, +1 TSS) **(b)** Colony PCR confirmation of deletion of the hairpin in P_{BMO} promoter on 1.6% agarose gel lane 1 – 1 kb+ DNA ladder (NEB); lane 2 – P_{BMO} WT promoter (413 bp); lane 3 – P_{BMO} Δhairpin (376 bp).

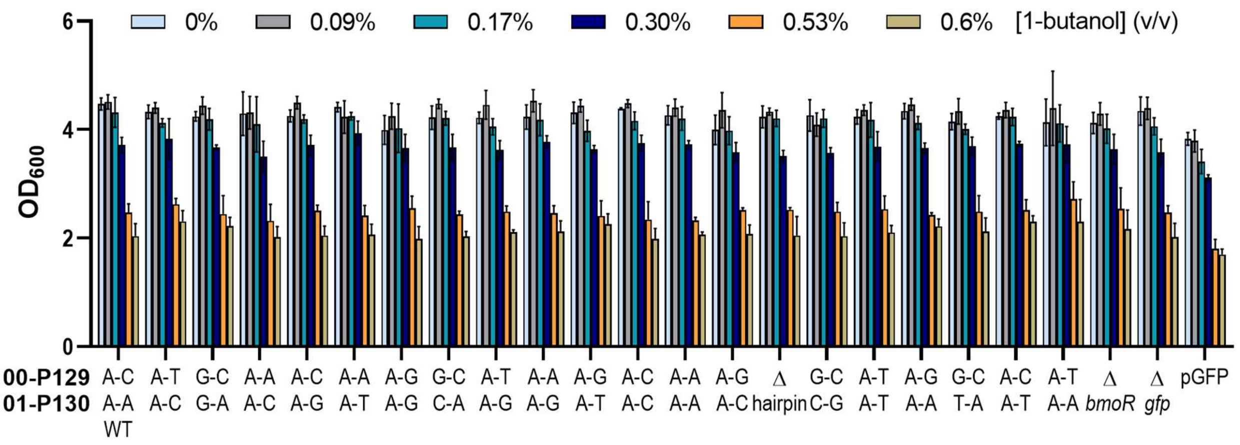

Supplementary Figure 2. OD_{600} of P_{BMO} hairpin mutants. Cell density decrease as the 1-butanol concentration increases.

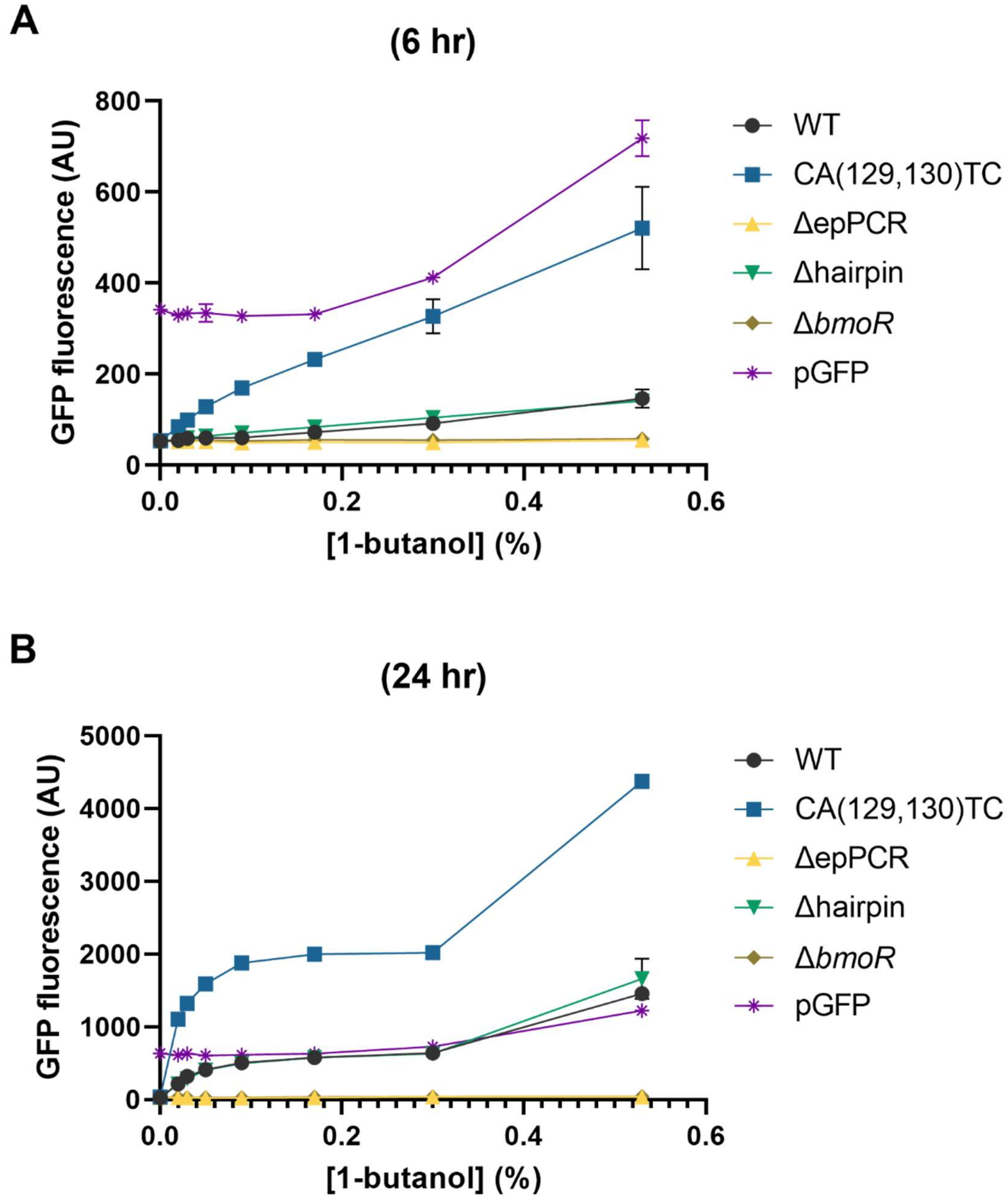

Supplementary Figure 3. Response curves of P_{BMO} hairpin mutants at **(a)** 6 hours and **(b)** 24 hours post-butanol induction.

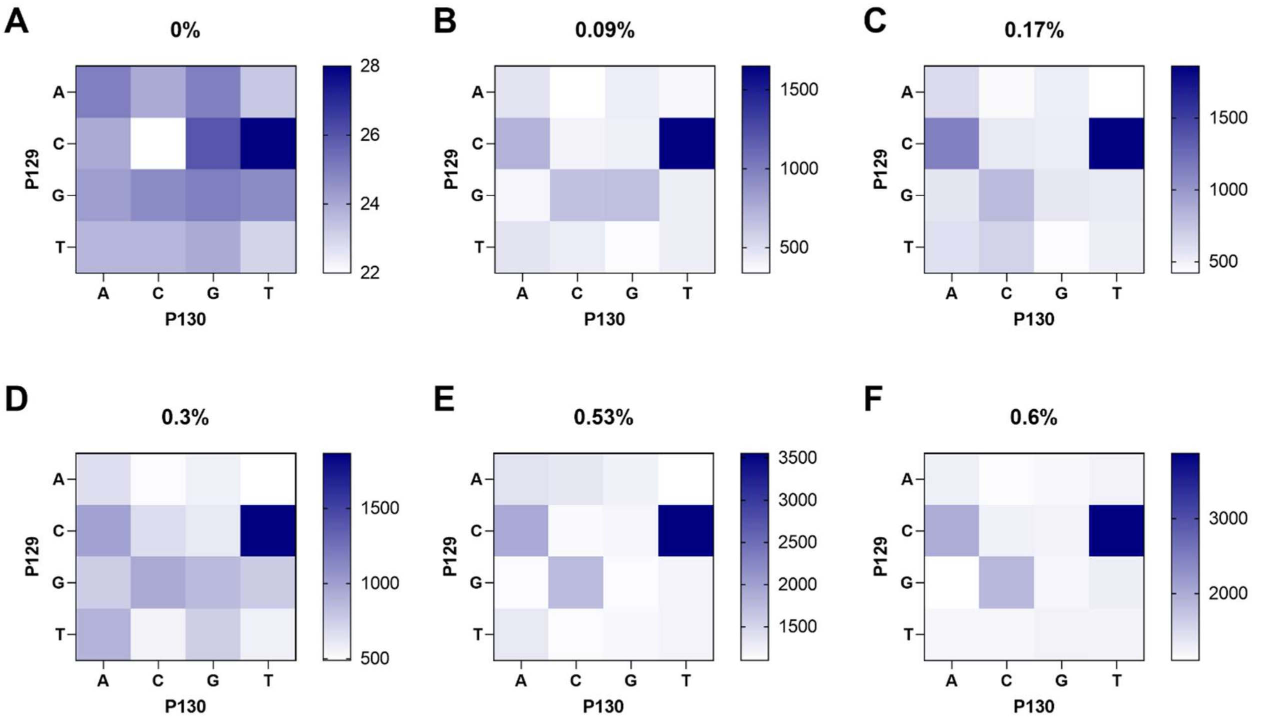

Supplementary Figure 4. Heat maps of the GFP fluorescence of site-specific mutations at P129 and P130 in P_{BMO} hairpin mutants. Site-specific dependence of P_{BMO} hairpin mutation sites P129 and P130 on the right half of the fifteen PBMO hairpin mutants and the wild-type hairpin when induced with **(a)** 0%, **(b)** 0.09%, **(c)** 0.17%, **(d)** 0.3%, **(e)** 0.53%, and **(f)** 0.6% 1-butanol (v/v). Two-way ANOVA.

Supplementary Tables

Supplementary Table 1. Mutational Bias Analysis of the unsorted P_{BMO} library (R=A/G, Y=T/C).

Mutation Types		Mutation Frequency
**Transition**	**R→R, Y→Y**	56.4%
	A→G, T→C	18.0%
	G→A, C→T	38.4%
**Transversion**	**R→Y, Y→R**	43.6%
	A→T, T→A	16.4%
	A→C, T→G	2.3%
	G→C, C→G	6.5%
	G→T, C→A	18.4%

Supplementary Table 2. Bacterial strains and plasmids used in this study.

**Plasmid**	**Description**	**Strain**	**Purpose**	**Source Reference**
$P_{SELECT\#1}$	P_{BmoR}-*bmoR*, P_{BMO}-*tetA*-*gfp*	*E. coli* (DH1α)		(Dietrich, Shis et al. 2013)
$P_{SELECT\#2}$	P_{BmoR}-*bmoR*, P_{BMO}-*tetA*	*E. coli* (DH1α)		(Dietrich, Shis et al. 2013)
WTΔ*bmoR*	ΔP_{BmoR}-*bmoR,* P_{BMO}-*gfp*	*E. coli* (NEB5α)	negative control	This study
WTΔ*gfp*	P_{BmoR}-*bmoR*, P_{BMO}-Δ*gfp*	*E. coli* (NEB5α)	negative control	This study
Δhairpin	P_{BmoR}-*bmoR*, P_{BMO}Δhairpin-*gfp*	*E. coli* (NEB5α)	hairpin characterizatio n	This study
ΔepPCR	P_{BmoR}-*bmoR*, P_{BMO}Δ247-nt-*gfp*	*E. coli* (NEB5α)	negative control	This study
pGFP	pUC19 vector; LacI-P_{lac}-*gfp*	*E. coli* (NEB5α)	positive control	This study
CA(129,130)TCΔ*bmoR*	ΔP_{BmoR}-*bmoR*, P_{BMO}-*gfp* with A_{100} A_{101} T_{129} C_{130}	*E. coli* (NEB5α)	negative control	This study
WT	P_{BmoR}-*bmoR*, P_{BMO}-*gfp*	*E. coli* (NEB5α)	wild-type control	This study
CA(129,130)AA	P_{BmoR}-*bmoR*, P_{BMO}-*gfp* with A_{100} A_{101} A_{129} A_{130}	*E. coli* (NEB5α)	P129, P130 characterizatio n	This study

CA(129,130)AC	P_{BmoR}-*bmoR*, P_{BMO}-*gfp* with A_{100} A_{101} A_{129} C_{130}	*E. coli* (NEB5α)	P129, P130 characterization	This study
CA(129,130)AG	P_{BmoR}-*bmoR*, P_{BMO}-*gfp* with A_{100} A_{101} A_{129} G_{130}	*E. coli* (NEB5α)	P129, P130 characterization	This study
CA(129,130)AT	P_{BmoR}-*bmoR*, P_{BMO}-*gfp* with A_{100} A_{101} A_{129} T_{130}	*E. coli* (NEB5α)	P129, P130 characterization	This study
CA(129,130)CC	P_{BmoR}-*bmoR*, P_{BMO}-*gfp* with A_{100} A_{101} C_{129} C_{130}	*E. coli* (NEB5α)	P129, P130 characterization	This study
CA(129,130)CG	P_{BmoR}-*bmoR*, P_{BMO}-*gfp* with A_{100} A_{101} C_{129} G_{130}	*E. coli* (NEB5α)	P129, P130 characterization	This study
CA(129,130)CT	P_{BmoR}-*bmoR*, P_{BMO}-*gfp* with A_{100} A_{101} C_{129} T_{130}	*E. coli* (NEB5α)	P129, P130 characterization	This study
CA(129,130)GA	P_{BmoR}-*bmoR*, P_{BMO}-*gfp* with A_{100} A_{101} G_{129} A_{130}	*E. coli* (NEB5α)	P129, P130 characterization	This study
CA(129,130)GC	P_{BmoR}-*bmoR*, P_{BMO}-*gfp* with A_{100} A_{101} G_{129} C_{130}	*E. coli* (NEB5α)	P129, P130 characterization	This study
CA(129,130)GG	P_{BmoR}-*bmoR*, P_{BMO}-*gfp* with A_{100} A_{101} G_{129} G_{130}	*E. coli* (NEB5α)	P129, P130 characterization	This study
CA(129,130)GT	P_{BmoR}-*bmoR*, P_{BMO}-*gfp* with A_{100} A_{101} G_{129} T_{130}	*E. coli* (NEB5α)	P129, P130 characterization	This study
CA(129,130)TA	P_{BmoR}-*bmoR*, P_{BMO}-*gfp* with A_{100} A_{101} T_{129} A_{130}	*E. coli* (NEB5α)	P129, P130 characterization	This study
CA(129,130)TC	P_{BmoR}-*bmoR*, P_{BMO}-*gfp* with A_{100} A_{101} T_{129} C_{130}	*E. coli* (NEB5α)	P129, P130 characterization	This study
CA(129,130)TG	P_{BmoR}-*bmoR*, P_{BMO}-*gfp* with A_{100} A_{101} T_{129} G_{130}	*E. coli* (NEB5α)	P129, P130 characterization	This study
CA(129,130)TT	P_{BmoR}-*bmoR*, P_{BMO}-*gfp* with A_{100} A_{101} T_{129} T_{130}	*E. coli* (NEB5α)	P129, P130 characterization	This study
AA(100,101)TG	P_{BmoR}-*bmoR*, P_{BMO}-*gfp* with T_{100} G_{101} C_{129} A_{130}	*E. coli* (NEB5α)	P129, P130 characterization	This study
AA(100,101)CG	P_{BmoR}-*bmoR*, P_{BMO}-*gfp* with C_{100} G_{101} C_{129} A_{130}	*E. coli* (NEB5α)	P129, P130 characterization	This study
AA(100,101)GG	P_{BmoR}-*bmoR*, P_{BMO}-*gfp* with G_{100} G_{101} C_{129} A_{130}	*E. coli* (NEB5α)	P129, P130 characterization	This study

AACA(100,101,129,130) CGCG	P_{BmoR}-*bmoR*, P_{BMO}-*gfp* with C_{100} G_{101} C_{129} G_{130}	*E. coli* (NEB5α)	P129, P130 characterizatio n	This study
AACA(100,101,129,130) GATC	P_{BmoR}-*bmoR*, P_{BMO}-*gfp* with G_{100} A_{101} T_{129} C_{130}	*E. coli* (NEB5α)	P129, P130 characterizatio n	This study
WT_{FLIP}	P_{BmoR}-*bmoR*, P_{BMO}-*gfp* with T_{100} G_{101} T_{129} T_{130}	*E. coli* (NEB5α)	P129, P130 characterizatio n	This study
CA(129,130)TC_{FLIP}	P_{BmoR}-*bmoR*, P_{BMO}-*gfp* with G_{100} A_{101} T_{129} T_{130}	*E. coli* (NEB5α)	P129, P130 characterizatio n	This study
G(21)A	P_{BmoR}-*bmoR*, P_{BMO}-*gfp* with A_{21}	*E. coli* (NEB5α)	Validation	This study
C(32)G	P_{BmoR}-*bmoR*, P_{BMO}-*gfp* with G_{21}	*E. coli* (NEB5α)	Validation	This study
G(48)C	P_{BmoR}-*bmoR*, P_{BMO}-*gfp* with C_{48}	*E. coli* (NEB5α)	Validation	This study
T(52)G	P_{BmoR}-*bmoR*, P_{BMO}-*gfp* with G_{52}	*E. coli* (NEB5α)	Validation	This study
G(77)C	P_{BmoR}-*bmoR*, P_{BMO}-*gfp* with C_{77}	*E. coli* (NEB5α)	Validation	This study
C(129)T	P_{BmoR}-*bmoR*, P_{BMO}-*gfp* with T_{129}	*E. coli* (NEB5α)	Validation	This study
A(130)C	P_{BmoR}-*bmoR*, P_{BMO}-*gfp* with C_{130}	*E. coli* (NEB5α)	Validation	This study
T(131)G	P_{BmoR}-*bmoR*, P_{BMO}-*gfp* with G_{131}	*E. coli* (NEB5α)	Validation	This study
C(133)T	P_{BmoR}-*bmoR*, P_{BMO}-*gfp* with T_{133}	*E. coli* (NEB5α)	Validation	This study
T(135)G	P_{BmoR}-*bmoR*, P_{BMO}-*gfp* with G_{135}	*E. coli* (NEB5α)	Validation	This study
C(162)G	P_{BmoR}-*bmoR*, P_{BMO}-*gfp* with G_{162}	*E. coli* (NEB5α)	Validation	This study
C(185)G	P_{BmoR}-*bmoR*, P_{BMO}-*gfp* with G_{185}	*E. coli* (NEB5α)	Validation	This study
G(186)C	P_{BmoR}-*bmoR*, P_{BMO}-*gfp* with C_{186}	*E. coli* (NEB5α)	Validation	This study
G(188)T	P_{BmoR}-*bmoR*, P_{BMO}-*gfp* with G_{188}	*E. coli* (NEB5α)	Validation	This study
C(190)T	P_{BmoR}-*bmoR*, P_{BMO}-*gfp* with T_{190}	*E. coli* (NEB5α)	IHF	This study
C(193)G	P_{BmoR}-*bmoR*, P_{BMO}-*gfp* with G_{193}	*E. coli* (NEB5α)	IHF	This study
G(196)A	P_{BmoR}-*bmoR*, P_{BMO}-*gfp* with A_{196}	*E. coli* (NEB5α)	Validation	This study
G(200)A	P_{BmoR}-*bmoR*, P_{BMO}-*gfp* with A_{200}	*E. coli* (NEB5α)	Validation	This study

A(202)T	P_{BmoR}-*bmoR*, P_{BMO}-*gfp* with T_{202}	*E. coli* (NEB5α)	IHF	This study
G(205)A	P_{BmoR}-*bmoR*, P_{BMO}-*gfp* with A_{205}	*E. coli* (NEB5α)	Validation	This study
GG(186,188)CT	P_{BmoR}-*bmoR*, P_{BMO}-*gfp* with C_{186} T_{188}	*E. coli* (NEB5α)	Validation	This study
TCT(131,133,135)GTG	P_{BmoR}-*bmoR*, P_{BMO}-*gfp* with G_{131} T_{133} G_{135}	*E. coli* (NEB5α)	Validation	This study
CA(129,130)TC//G(21)A	P_{BmoR}-*bmoR*, P_{BMO}-*gfp* with A_{100} A_{101} T_{129} C_{130} A_{21}	*E. coli* (NEB5α)	Validation	This study
CA(129,130)TC//C(32)G	P_{BmoR}-*bmoR*, P_{BMO}-*gfp* with A_{100} A_{101} T_{129} C_{130} G_{32}	*E. coli* (NEB5α)	Validation	This study
CA(129,130)TC//G(48)C	P_{BmoR}-*bmoR*, P_{BMO}-*gfp* with A_{100} A_{101} T_{129} C_{130} C_{48}	*E. coli* (NEB5α)	Validation	This study
CA(129,130)TC//T(52)G	P_{BmoR}-*bmoR*, P_{BMO}-*gfp* with A_{100} A_{101} T_{129} C_{130} G_{52}	*E. coli* (NEB5α)	Validation	This study
CA(129,130)TC//G(77)C	P_{BmoR}-*bmoR*, P_{BMO}-*gfp* with A_{100} A_{101} T_{129} C_{130} C_{77}	*E. coli* (NEB5α)	Validation	This study
CA(129,130)TC//T(131)G	P_{BmoR}-*bmoR*, P_{BMO}-*gfp* with A_{100} A_{101} T_{129} C_{130} G_{131}	*E. coli* (NEB5α)	Validation	This study
CA(129,130)TC//C(133)T	P_{BmoR}-*bmoR*, P_{BMO}-*gfp* with A_{100} A_{101} T_{129} C_{130} T_{133}	*E. coli* (NEB5α)	Validation	This study
CA(129,130)TC//T(135)G	P_{BmoR}-*bmoR*, P_{BMO}-*gfp* with A_{100} A_{101} T_{129} C_{130} G_{135}	*E. coli* (NEB5α)	Validation	This study
CA(129,130)TC//C(162)G	P_{BmoR}-*bmoR*, P_{BMO}-*gfp* with A_{100} A_{101} T_{129} C_{130} G_{162}	*E. coli* (NEB5α)	Validation	This study
CA(129,130)TC//C(185)G	P_{BmoR}-*bmoR*, P_{BMO}-*gfp* with A_{100} A_{101} T_{129} C_{130} G_{185}	*E. coli* (NEB5α)	Validation	This study
CA(129,130)TC//G(186)C	P_{BmoR}-*bmoR*, P_{BMO}-*gfp* with A_{100} A_{101} T_{129} C_{130} C_{186}	*E. coli* (NEB5α)	Validation	This study
CA(129,130)TC//G(188)T	P_{BmoR}-*bmoR*, P_{BMO}-*gfp* with A_{100} A_{101} T_{129} C_{130} T_{188}	*E. coli* (NEB5α)	Validation	This study
CA(129,130)TC//G(196)A	P_{BmoR}-*bmoR*, P_{BMO}-*gfp* with A_{100} A_{101} T_{129} C_{130} A_{196}	*E. coli* (NEB5α)	Validation	This study
CA(129,130)TC//G(200)A	P_{BmoR}-*bmoR*, P_{BMO}-*gfp* with A_{100} A_{101} T_{129} C_{130} A_{200}	*E. coli* (NEB5α)	Validation	This study
CA(129,130)TC//G(205)A	P_{BmoR}-*bmoR*, P_{BMO}-*gfp* with A_{100} A_{101} T_{129} C_{130} A_{205}	*E. coli* (NEB5α)	Validation	This study
CA(129,130)TC//G,G(186,188)C,T	P_{BmoR}-*bmoR*, P_{BMO}-*gfp* with A_{100} A_{101} T_{129} C_{130} C_{186} T_{188}	*E. coli* (NEB5α)	Validation	This study
CA(129,130)TC//T,C,T(131,133,135)G,T,G	P_{BmoR}-*bmoR*, P_{BMO}-*gfp* with A_{100} A_{101} T_{129} G_{131} T_{133} G_{135}	*E. coli* (NEB5α)	Validation	This study
Δhairpin//G(21)A	P_{BmoR}-*bmoR*, P_{BMO} Δhairpin-*gfp* with A_{21}	*E. coli* (NEB5α)	Validation	This study
Δhairpin//C(32)G	P_{BmoR}-*bmoR*, P_{BMO} Δhairpin-*gfp* with G_{32}	*E. coli* (NEB5α)	Validation	This study
Δhairpin//G(48)C	P_{BmoR}-*bmoR*, P_{BMO} Δhairpin-*gfp* with C_{48}	*E. coli* (NEB5α)	Validation	This study
Δhairpin//T(52)G	P_{BmoR}-*bmoR*, P_{BMO} Δhairpin-*gfp* with G_{52}	*E. coli* (NEB5α)	Validation	This study

Δhairpin//G(77)C	P_{BmoR}-*bmoR*, P_{BMO} Δhairpin-*gfp* with C_{77}	*E. coli* (NEB5α)	Validation	This study
Δhairpin//C(162)G	P_{BmoR}-*bmoR*, P_{BMO} Δhairpin-*gfp* with G_{162}	*E. coli* (NEB5α)	Validation	This study
Δhairpin//C(185)G	P_{BmoR}-*bmoR*, P_{BMO} Δhairpin-*gfp* with G_{185}	*E. coli* (NEB5α)	Validation	This study
Δhairpin//G(186)C	P_{BmoR}-*bmoR*, P_{BMO} Δhairpin-*gfp* with C_{186}	*E. coli* (NEB5α)	Validation	This study
Δhairpin//G(188)T	P_{BmoR}-*bmoR*, P_{BMO} Δhairpin-*gfp* with T_{188}	*E. coli* (NEB5α)	Validation	This study
Δhairpin//G(196)A	P_{BmoR}-*bmoR*, P_{BMO} Δhairpin-*gfp* with A_{196}	*E. coli* (NEB5α)	Validation	This study
Δhairpin//G(200)A	P_{BmoR}-*bmoR*, P_{BMO} Δhairpin-*gfp* with A_{200}	*E. coli* (NEB5α)	Validation	This study
Δhairpin//G(205)A	P_{BmoR}-*bmoR*, P_{BMO} Δhairpin-*gfp* with A_{205}	*E. coli* (NEB5α)	Validation	This study
Δhairpin//G,G(186,188)C,T	P_{BmoR}-*bmoR*, P_{BMO} Δhairpin-*gfp* with C_{186} C_{188}	*E. coli* (NEB5α)	Validation	This study
IHF-5'-half	P_{BmoR}-*bmoR*, P_{BMO}-*gfp* with $GCGTTCGCTCCCGCGGC_{174\text{-}190}$	*E. coli* (NEB5α)	IHF	This study
IHF-3'-half	P_{BmoR}-*bmoR*, P_{BMO}-*gfp* with $GCCGCGGGTGTACCGTT_{191\text{-}207}$	*E. coli* (NEB5α)	IHF	This study
IHF-5' & 3'	P_{BmoR}-*bmoR*, P_{BMO}-*gfp* with $GCGTTCGCTCCCGCGGCGCCGCGGGTGTACCGTT_{174\text{-}207}$	*E. coli* (NEB5α)	IHF	This study
IHF-pspA	P_{BmoR}-*bmoR*, P_{BMO}-*gfp* with $TCAATCAGATCTTTATAAATCAAAAAGATAAAAA_{174\text{-}207}$	*E. coli* (NEB5α)	IHF	This study
IHF-glnHp2	P_{BmoR}-*bmoR*, P_{BMO}-*gfp* with $TTTGCCGCATCTCGAAAAATCAATGAATTACTCG_{174\text{-}207}$	*E. coli* (NEB5α)	IHF	This study
IHF-λH'	P_{BmoR}-*bmoR*, P_{BMO}-*gfp* with $GCCAAAAAAGCATTGCTTATCAATTTGTTGCACC_{174\text{-}207}$	*E. coli* (NEB5α)	IHF	This study

Supplementary Table 3. Primers used to construct plasmids in this study. Mutations and overhangs hifi assembly primer regions are in lower case letters.

Plasmid	Name	Forward Primer (5'-->3')	Name	Reverse Primer (5'-->3')
WTΔ*bmoR*	NK155	CCACAGATAGTAGGTGCTG	NK212	AGGTGGCACTTTTCGGG
WTΔ*gfp*	NK107	CTACAAATAAGGATCCTAACTCGAGTCTAGACC	NK129	TTGTGTGTTCTGCTGTCGGTAG
Δhairpin	NK218	TTGCCACACCCAACCGGA	NK219	AATCTTCCGCGCTGTCCG
ΔepPCR	NK210	TCTTTAACGTGTAACACACG	NK160	GACCTTGAGGTGACCTTG
CA(129,130)TCΔ*bmoR*	NK155	CCACAGATAGTAGGTGCTG	NK212	AGGTGGCACTTTTCGGG
WTΔbmoR	NK155	CCACAGATAGTAGGTGCTG	NK212	AGGTGGCACTTTTCGGG
pGFP	NK109	ctttactcatAGCTGTTTCCTGTGTGAAATTGTTATCC	NK110	ctacaaataaAAGGGCCTCGTGATACGCCT
	NK111	cgaggcccttTTATTTGTAGAGCTCATCCATG	NK112	ggaaacagctATGAGTAAAGGAGAAGAACTTTTC
CA(129,130)AA	NK1	CCTCGGGCTGaaTCCTTGCCAC	NK16	CACGCACGCTCGGGCTGT
CA(129,130)AC	NK2	CCTCGGGCTGacTCCTTGCCACACCC	NK16	CACGCACGCTCGGGCTGT
CA(129,130)AG	NK3	CCTCGGGCTGagTCCTTGCCACACCCAAC	NK16	CACGCACGCTCGGGCTGT
CA(129,130)AT	NK4	CCTCGGGCTGatTCCTTGCCACACCC	NK16	CACGCACGCTCGGGCTGT
CA(129,130)CC	NK5	CCTCGGGCTGccTCCTTGCCAC	NK16	CACGCACGCTCGGGCTGT
CA(129,130)CG	NK6	CCTCGGGCTGcgTCCTTGCCAC	NK16	CACGCACGCTCGGGCTGT
CA(129,130)CT	NK7	CCTCGGGCTGctTCCTTGCCAC	NK16	CACGCACGCTCGGGCTGT
CA(129,130)GA	NK8	CCTCGGGCTGgaTCCTTGCCAC	NK16	CACGCACGCTCGGGCTGT
CA(129,130)GC	NK9	CCTCGGGCTGgcTCCTTGCCACACCC	NK16	CACGCACGCTCGGGCTGT
CA(129,130)GG	NK10	CCTCGGGCTGggTCCTTGCCACACCCAAC	NK16	CACGCACGCTCGGGCTGT
CA(129,130)GT	NK11	CCTCGGGCTGgtTCCTTGCCACACCC	NK16	CACGCACGCTCGGGCTGT
CA(129,130)TA	NK12	CCTCGGGCTGtaTCCTTGCCAC	NK16	CACGCACGCTCGGGCTGT
CA(129,130)TC	NK13	CCTCGGGCTGtcTCCTTGCCACACCCAAC	NK16	CACGCACGCTCGGGCTGT
CA(129,130)TG	NK14	CCTCGGGCTGtgTCCTTGCCACACCCAAC	NK16	CACGCACGCTCGGGCTGT
CA(129,130)TT	NK15	CCTCGGGCTGttTCCTTGCCACACCCAAC	NK16	CACGCACGCTCGGGCTGT
AA(100,101)TG	NK119	GAAGATTGGAtgCAGCCCGAGCGTGCGTGCC	NK121	CGCGCTGTCCGCCGAGGT
AA(100,101)CG	NK120	GAAGATTGGAcgCAGCCCGAGCGTGCGTGCCTC	NK121	CGCGCTGTCCGCCGAGGT
AA(100,101)GG	NK123	GAAGATTGGANNCAGCCCGAGCGTGCGTGCCTC	NK121	CGCGCTGTCCGCCGAGGT
AACA(100,101,129,130)CGCG	NK120	GAAGATTGGAcgCAGCCCGAGCGTGCGTGCCTC	NK121	CGCGCTGTCCGCCGAGGT

AACA(100,101,129,130)GATC	NK331	gtgcctcgggctgtcTCCTTGCCACACCCAACC	NK332	gcacgctcgggctgtcTCCAATCTTCCGCGCTGTC
WT_{FLIP}	NK292	cacgctcgggctgtttccTTGCCACACCCAACCGGA	NK293	cgtgcctcgggctgcatccAATCTTCCGCGCTGTCCG
$CA(129,130)TC_{FLIP}$	NK292	cacgctcgggctgtttccTTGCCACACCCAACCGGA	NK294	cgtgcctcgggctgtctccAATCTTCCGCGCTGTCCG
G(21)A	NK226	aGCTGCTCATGCTCCTGTCGC	NK241	GCAGCACCTACTATCTGTGGG
C(32)G	NK227	GGCTGCTCATGgTCCTGTCGC	NK241	GCAGCACCTACTATCTGTGGG
G(48)C	NK228	GGTAGCcCGCTGTTACGCGACC	NK242	GCGACAGGAGCATGAGCAG
T(52)G	NK229	GGTAGCGCGCgGTTACGCGAC	NK242	GCGACAGGAGCATGAGCAG
G(77)C	NK230	CCCGGACCTCcGCGGACAGCG	NK243	GGCGGTCGCGTAACAGCG
C(129)T	NK12	CCTCGGGCTGtaTCCTTGCCAC	NK16	CACGCACGCTCGGGCTGT
A(130)C	NK05	CCTCGGGCTGccTCCTTGCCAC	NK16	CACGCACGCTCGGGCTGT
T(131)G	NK231	GGGCTGCAgCCTTGCCACACC	NK244	GAGGCACGCACGCTCGGG
C(133)T	NK232	GGGCTGCATCtTTGCCACACCC	NK244	GAGGCACGCACGCTCGGG
T(135)G	NK233	GGGCTGCATCCTgGCCACACCC	NK244	GAGGCACGCACGCTCGGG
C(162)G	NK234	TTCGTCGGACgGCTCGACATTCGC	NK245	TCCGGTTGGGTGTGGCAAG
C(185)G	NK235	CCGCcGGAGCGAACGCGAATGTC	NK246	CGCCGCGGGTGTACCGTTG
G(186)C	NK236	CCGgGGGAGCGAACGCGAATGTC	NK246	CGCCGCGGGTGTACCGTTG
G(188)T	NK237	CaGCGGGAGCGAACGCGAATGTC	NK246	CGCCGCGGGTGTACCGTTG
C(190)T	NK325	gccgcgggtgtaccgttGCGTTACAGATGTACCCTTC	NK326	accgcgggagcgaacgcGAATGTCGAGCGGTCCGA
C(193)G	NK327	gcggcgggtgtaccgttGCGTTACAGATGTACCCTTC	NK328	gccgcgggagcgaacgcGAATGTCGAGCGGTCCGA
G(196)A	NK238	CGCCGCaGGTGTACCGTTGCG	NK247	CCGCGGGAGCGAACGCG
G(200)A	NK239	CGCCGCGGGTaTACCGTTGCG	NK247	CCGCGGGAGCGAACGCG
A(202)T	NK329	gccgcgggtgttccgttGCGTTACAGATGTACCCTTC	NK330	gccgcgggagcgaacgcGAATGTCGAGCGGTCCGA
G(205)A	NK240	CGCCGCGGGTGTACCaTTGCGTTACAG	NK247	CCGCGGGAGCGAACGCG
GG(186,188)CT	NK253	CaGgGGGAGCGAACGCGAATGTC	NK246	CGCCGCGGGTGTACCGTTG
TCT(131,133,135)GTG	NK254	TCGGGCTGCAgCtTgGCCACACCCAACC	NK252	GGCACGCACGCTCGGGCT
CA(129,130)TC//G(21)A	NK13	CCTCGGGCTGtcTCCTTGCCACACCCAAC	NK16	CACGCACGCTCGGGCTGT
CA(129,130)TC//C(32)G	NK13	CCTCGGGCTGtcTCCTTGCCACACCCAAC	NK16	CACGCACGCTCGGGCTGT
CA(129,130)TC//G(48)C	NK13	CCTCGGGCTGtcTCCTTGCCACACCCAAC	NK16	CACGCACGCTCGGGCTGT
CA(129,130)TC//T(52)G	NK13	CCTCGGGCTGtcTCCTTGCCACACCCAAC	NK16	CACGCACGCTCGGGCTGT

CA(129,130)TC//G(77)C	NK13	CCTCGGGCTGtcTCCTTGCCACACCCAAC	NK16	CACGCACGCTCGGGCTGT
CA(129,130)TC//T(131)G	NK248	GGGCTGTCgCCTTGCCACACC	NK244	GAGGCACGCACGCTCGGG
CA(129,130)TC//C(133)T	NK249	GGGCTGTCTCtTTGCCACACCC	NK244	GAGGCACGCACGCTCGGG
CA(129,130)TC//T(135)G	NK250	GGGCTGTCTCCTgGCCACACCC	NK244	GAGGCACGCACGCTCGGG
CA(129,130)TC//C(162)G	NK13	CCTCGGGCTGtcTCCTTGCCACACCCAAC	NK16	CACGCACGCTCGGGCTGT
CA(129,130)TC//C(185)G	NK13	CCTCGGGCTGtcTCCTTGCCACACCCAAC	NK16	CACGCACGCTCGGGCTGT
CA(129,130)TC//G(186)C	NK13	CCTCGGGCTGtcTCCTTGCCACACCCAAC	NK16	CACGCACGCTCGGGCTGT
CA(129,130)TC//G(188)T	NK13	CCTCGGGCTGtcTCCTTGCCACACCCAAC	NK16	CACGCACGCTCGGGCTGT
CA(129,130)TC//G(196)A	NK13	CCTCGGGCTGtcTCCTTGCCACACCCAAC	NK16	CACGCACGCTCGGGCTGT
CA(129,130)TC//G(200)A	NK13	CCTCGGGCTGtcTCCTTGCCACACCCAAC	NK16	CACGCACGCTCGGGCTGT
CA(129,130)TC//G(205)A	NK13	CCTCGGGCTGtcTCCTTGCCACACCCAAC	NK16	CACGCACGCTCGGGCTGT
CA(129,130)TC//G,G(186,188)C,T	NK13	CCTCGGGCTGtcTCCTTGCCACACCCAAC	NK16	CACGCACGCTCGGGCTGT
CA(129,130)TC//T,C, T(131,133,135)G,T,G	NK251	TCGGGCTGTCgCtTgGCCACACCCAACC	NK252	GGCACGCACGCTCGGGCT
Δhairpin//G(21)A	NK226	aGCTGCTCATGCTCCTGTCGC	NK241	GCAGCACCTACTATCTGTGGG
Δhairpin//C(32)G	NK227	GGCTGCTCATGgTCCTGTCGC	NK241	GCAGCACCTACTATCTGTGGG
Δhairpin//G(48)C	NK228	GGTAGCcCGCTGTTACGCGACC	NK242	GCGACAGGAGCATGAGCAG
Δhairpin//T(52)G	NK229	GGTAGCGCGCgGTTACGCGAC	NK242	GCGACAGGAGCATGAGCAG
Δhairpin//G(77)C	NK230	CCCGGACCTCcGCGGACAGCG	NK243	GGCGGTCGCGTAACAGCG
Δhairpin//C(162)G	NK234	TTCGTCGGACgGCTCGACATTCGC	NK245	TCCGGTTGGGTGTGGCAAG
Δhairpin//C(185)G	NK235	CCGCcGGAGCGAACGCGAATGTC	NK246	CGCCGCGGGTGTACCGTTG
Δhairpin//G(186)C	NK236	CCGgGGGAGCGAACGCGAATGTC	NK246	CGCCGCGGGTGTACCGTTG
Δhairpin//G(188)T	NK237	CaGCGGGAGCGAACGCGAATGTC	NK246	CGCCGCGGGTGTACCGTTG
Δhairpin//G(196)A	NK238	CGCCGCaGGTGTACCGTTGCG	NK247	CCGCGGGAGCGAACGCG
Δhairpin//G(200)A	NK239	CGCCGCGGGTaTACCGTTGCG	NK247	CCGCGGGAGCGAACGCG
Δhairpin//G(205)A	NK240	CGCCGCGGGTGTACCaTTGCGTTACAG	NK247	CCGCGGGAGCGAACGCG
Δhairpin//G,G(186,188)C,T	NK253	CaGgGGGAGCGAACGCGAATGTC	NK246	CGCCGCGGGTGTACCGTTG
IHF-5'-half	NK319	aatcaaggtgttccattGCGTTACAGATGTACCCTTC	NK320	gccgcgggagcgaacgcGAATGTCGAGCGGTCCGA
IHF-3'-half	NK321	gccgcgggtgtaccgttGCGTTACAGATGTACCCTTC	NK322	ataaggatatcgaacccGAATGTCGAGCGGTCCGA
IHF-5' & 3'	NK323	aatcaaggtgttccattGCGTTACAGATGTACCCTTC	NK324	ataaggatatcgaacccGAATGTCGAGCGGTCCGA

IHF-pspA	NK313	aatcaaaaagataaaaaGCGT TACAGATGTACCCTTC	NK314	tataaagatctgattgaGAATG TCGAGCGGTCCGA
IHF-glnHp2	NK315	aatcaatgaattactcgGCGT TACAGATGTACCCTTC	NK316	tttcgagatgcggcaaaGAATG TCGAGCGGTCCGA
IHF-λH'	NK317	tatcaatttgttgcaccGCGT TACAGATGTACCCTTC	NK318	agcaatgctttttggcGAATG TCGAGCGGTCCGA

Supplementary Table 4. Library generation, Sanger & NGS, and Additional Primers. Mutations and overhangs HiFi assembly primer regions are in lower case letters.

Name	Name	Forward Primer (5'-->3')	Name	Reverse Primer (5'-->3')
Sanger sequencing primer 1			NK22	GCATTGAACACCATAAGAGAAAGTA GTGACA
Sanger sequencing primer 2			NK129	TTGTGTGTTCTGCTGTCGGTAG
correcting BmoR Alanine->Valine 31	NK222	GACATCAGCCaCTTTCCCCGG	NK223	GTTTTGCGCTCGTGGACC
epPCR library generation	NK161	ctcaaggtcacctcaaggtcCC ACAGATAGTAGGTGCTG	NK211	ggcgtgtgttacacgttaaagaAGG GTACATCTGTAACGC
epPCR library Vector	NK210	TCTTTAACGTGTAACACACG	NK160	GACCTTGAGGTGACCTTG
Amplicon Illumina partial adapters	NK224	ACACTCTTTCCCTACACGACGC TCTTCCGATCTccacagatagt aggtgctg	NK225	GACTGGAGTTCAGACGTGTGCTCTT CCGATCTggcgtgtgttacacgtta aag

References

Dietrich, J. A., D. L. Shis, A. Alikhani and J. D. Keasling (2013). "Transcription factor-based screens and synthetic selections for microbial small-molecule biosynthesis." ACS synthetic biology **2**(1): 47-58.
